# Transition from somatic embryo to friable embryogenic callus in cassava: dynamic changes in cellular structure, physiological status, and gene expression profiles

**DOI:** 10.3389/fpls.2015.00824

**Published:** 2015-10-06

**Authors:** Qiuxiang Ma, Wenzhi Zhou, Peng Zhang

**Affiliations:** National Key Laboratory of Plant Molecular Genetics, Institute of Plant Physiology and Ecology, Shanghai Institutes for Biological Sciences, Chinese Academy of SciencesShanghai, China

**Keywords:** *Manihot esculenta* Crantz, somatic embryo, friable embryogenic callus, RNA-seq, somaclonal variation, DNA methylation, ploidy

## Abstract

Friable embryogenic callus (FEC) is considered as the most suitable material for efficient genetic transformation of cassava. Heavy genotype dependence of FEC induction and amenability to somaclonal variation limits the production and maintenance of reliable FEC. Identifying key elements involved in biological processes from somatic embryos (SEs) to FEC at different stages provides critical insights for FEC improvement. Cytological observation showed a dramatic change of subcellular structures among SEs, fresh FEC (FFEC), and old FEC (OFEC). Decrease of sucrose and increase of fructose and glucose were detected in OFEC. A total of 6871 differentially expressed genes (DEGs) were identified from SEs, FFEC, and OFEC by RNA-seq. Analysis of the DEGs showed that FEC induction was accompanied by the process of dedifferentiation, whereas the epigenetics modification occurred during the continuous subculturing process. The cell structure was reconstructed, mainly including the GO terms of “cell periphery” and “external encapsulating structure”; in parallel, the internal mechanisms changed correspondingly, including the biological process of glycolysis and metabolisms of alanine, aspartate, and glutamate. The significant reduction of genomic DNA methylation in OFEC indicated altered gene expression via chromatin modification. These results indicate that the induction and long-term subculture of FEC is a complicated biological process involving changes of genome modification, gene expression, and subcellular reconstruction. The findings will be useful for improving FEC induction and maintenance from farmer-preferred cassava cultivars recalcitrant to genetic transformation, hence improving cassava through genetic engineering.

## Introduction

Cassava (*Manihot esculenta* Crantz) is widely cultivated in tropical and subtropical regions as an important source of food, feed, and industrial materials (Nassar and Ortiz, 2009; Sayre et al., 2011; Beeching, 2013). Because of high genetic heterozygosity, few flowers, low pollen fertility and fruit set rate, self-incompatibility, and serious trait separation in progeny, the conventional breeding of cassava is seriously hindered (Ceballos et al., 2010, 2012). Genetic engineering is an alternative effective method used to introduce value-adding genes to modify important agronomic traits (Taylor et al., 2004; Liu et al., 2011; Sayre et al., 2011). The friable embryogenic callus (FEC)-based genetic transformation is reported to be the most successful system in this decade (Raemakers et al., 1996; Schöpke et al., 1996; Taylor et al., 1996, 2004; Zhang et al., 2000; Bull et al., 2009; Liu et al., 2011; Xu et al., 2013).

FEC induction in cassava is a complicated process compared with the transformation protocols of many other species (Zhang and Gruissem, 2004; Bull et al., 2009). The process includes several steps as follows: primary somatic embryo (SE) induction, secondary SE multiplication, fresh FEC (FFEC) induction, and FEC subculturing and multiplication (Zhang, 2000). In cassava, primary and secondary SEs are easily achieved by incubating explants such as young leaf lobes or isolated shoot apexes in a culture medium supplemented with a auxin-like herbicide such as picloram or 2,4-D. Under a suitable condition, FFEC clusters can be induced from secondary SEs by continuous incubation on a GD medium supplemented with 50 μM picloram, a process that is well-established and demonstrated in the model cultivar TMS60444 (Taylor et al., 1996; Bull et al., 2009). However, the FEC induction originating from SEs is a bottleneck and is greatly genotype dependent (Liu et al., 2011). Only recently, cultivars beyond TMS60444 could be used for FEC induction and genetic transformation, such as African farmer–preferred cultivars TME7, TME14, TME204, Ebwanatereka, Kibandameno, and Serere (Vanderschuren et al., 2012; Zainuddin et al., 2012; Chetty et al., 2013; Nyaboga et al., 2013, 2015; Chauhan et al., 2015). For Asian cultivars, the FECs of KU50, SC8, and SC205 were successfully induced in Shanghai Center for Cassava Biotechnology although the efficiency still needs to be improved in comparison with TMS60444. Nevertheless, severe somaclonal variation of regenerated plants from FECs after a short-term subculture (in 3 months) makes the process laborious and limits its application (Raemakers et al., 2001; Taylor et al., 2001, 2004; Bull et al., 2009). Understanding the regulatory mechanisms leading to the initiation of FEC from SEs and somaclonal variation in FEC is an essential step for improving the system.

Various factors including explant types, basic media, and hormones that affect SEs and the FEC induction process have been investigated by various tissue culture techniques (Reviewed by Liu et al., 2011). Recently, the studies optimizing the FEC transformation platform in several farmer-preferred cassava cultivars have made great progress (Zainuddin et al., 2012; Chetty et al., 2013; Chauhan et al., 2015). However, the changes of cellular structures and physiological status from SEs to the FEC culture process are largely unknown, and less regulatory information is available at the molecular level. Previous studies indicate that SEs of cassava have a multicellular origin, and embryogenic masses are formed around the procambium (Baba et al., 2008). It will be useful to identify some cues to analyze the possibility of FEC induction and to evaluate the potential of FEC transformation.

In cassava, the decrease in regeneration ability and the increase in abnormal morphology of regenerated plants prevent the usage of old FEC (OFEC) that has a long-term *in vitro* culturing process. Many reasons might induce the occurrence of somaclonal variation during the tissue culture process, including irregular cell cycle, DNA methylation ratio change, ploidy change, etc. (Fraga and Esteller, 2002; Chakrabarty et al., 2003; Bairu et al., 2011; Miguel and Marum, 2011; Rival et al., 2013). In cassava, the ploidy levels of *in vitro* plantlets originating from stem cuttings treated with colchicine and oryzalin or regenerated from cotyledons of cassava SEs were assessed by the flow cytometry technique (Awoleye et al., 1994; Hankoua et al., 2005). The origin of anther-derived calli was assessed in cassava for their ploidy levels (Perera et al., 2014). With developing second-generation sequencing technologies, using the transcriptomic studies of somatic embryogenesis to identify the key factors that change during somatic embryogenesis and callus induction has become a fast and useful practical tool in various plants, for example, maize, oil palm, and longan (Lin et al., 2009; Lai and Lin, 2013; Salvo et al., 2014).

To better understand the molecular changes in the process of cassava FEC induction and subculturing, the gene expression profiles of SEs, FFEC, and OFEC were characterized using the RNA-seq technology. The major pathways and key events related to these representative materials of FEC development were illustrated by the deep analysis of differentially expressed genes (DEGs). In combination with the histological and chemical analysis during the FEC formation process, the understanding of the molecular regulatory and networks regulating FEC formation and subculture process has increased, which could provide guidance toward improving FEC induction from recalcitrant cultivars as well as ensuring the quality of regenerated plants through improved culture conditions.

## Materials and methods

### Plant materials used for solexa sequencing

The cassava cultivar TMS60444 was used as a plant material for SEs and FEC induction according to the method described in a previous study (Zhang and Gruissem, 2004). Briefly, SEs were induced from shoot apical meristems and axillary buds on CIM (MS medium, Murashige and Skoog, 1962 with 2% sucrose and 2 μM CuSO4, 12 mg/l picloram, solidified with 0.6% agar, pH 5.8) in the dark and subcultured every 2 weeks. Four to six-week-old SEs were transferred onto the GD medium (GD salts and vitamins Gresshoff and Doy, 1974, 12 mg/l picloram, 2% sucrose, 0.5% agar, pH 5.8) at (26 ± 2)°C in dark for FFEC induction. When FFEC appeared, it was transferred onto a fresh GD medium and cultivated at (26 ± 2)°C with a 16 h photoperiod of weak light (~10 μmol/m^2^s) and subcultured at 3 week intervals. Two-week-old SEs cultured on a GD medium, FFEC emerging from SEs, and 9 month-old OFEC were used for analysis.

### Histological observation by cross section and transmission electronic microscopy (TEM)

The SE, FFEC, and OFEC samples were collected and fixed in formalin-acetic acid-alcohol (FAA, 10:5:50%) for 48 h at 4°C and then embedded in paraffin wax. Sections of 15 μm thickness were cut and placed on silane-coated slides to fix the samples. After drying overnight at 37°C, the samples were deparaffinized and stained with hematoxylin and eosin solution. TUNEL and DAPI were used to counter-stain nuclei before the last washing for 5 min. All pictures were taken using a fluorescent microscope (Olympus BX51, Olympus Optical, Tokyo, Japan) with differential interference contrast using appropriate filters. For TEM analysis, the fixed tissue was washed, and fixed with 1% OsO_4_ overnight at 4°C. After washing in phosphate buffer, the samples were dehydrated through a series of ethanol solutions, then infiltrated with a graded series of epoxy resin in epoxy propane, and embedded in Epon 812 resin. Thin sections were stained in 1% uranyl acetate, followed by lead citrate solution, and viewed with a transmission electron microscope (HITACHI H-7650, Hitachi, Tokyo, Japan).

### High-performance liquid chromatography (HPLC) analysis of monosaccharide content

Glucose, fructose, and sucrose were quantified using the HPLC–refractive index (HPLC-RI) system. Briefly, the frozen samples in liquid nitrogen were ground into a powder, and 100 mg of this sample was dissolved in 0.7 ml of 80% ethanol to extract the sugars. The sample was thoroughly vortexed and incubated for 2 h at 70°C. Aliquots of 0.7 ml of HPLC-grade water and 0.7 ml chloroform were added to the sample. After shaking the mixtures several times, they were centrifuged at 12,000 g for 10 min. Then, 0.7 ml of the aqueous supernatant was transferred into 1.5 ml Eppendorf tubes and 0.7 ml of chloroform was added. The samples were vortexed for 2–3 min. After centrifugation at 12,000 g for 10 min, 0.5 ml of the supernatant was transferred to a glass tube for HPLC analysis of each sugar component. The sugar-separation method used was that described by the manufacturer but with slight modifications; the HPLC column (ZORBAX carbohydrate column; 4.6 × 150 mm, 5 μm) of Agilent technologies with a differential refraction detector was used. The mobile phase consisted of 75% acetonitrile with a flow rate of 0.8 ml/min; the temperature of the column was maintained at 35°C. The sugars were identified based on the retention time of the standards, and sample concentrations were calculated from the external standard curve.

### Preparation of digital gene expression libraries and sequencing

The harvested samples from six petri dishes (30–40 tissue clusters/Petri dish) of SEs, FFEC, or OFEC were frozen in liquid nitrogen for homogeneity and held at −80°C prior to transporting to the Beijing Genome Institute (BGI, Shenzhen, China) for RNA extraction. Total RNA extraction, mRNA purification, cDNA synthesis, and RNA-seq library construction were done as described in a previous study (Liu et al., 2014). The Illumina Cluster Station and Illumina HiSeq 2000 System (Illumina, Inc., CA, USA) was used to perform sequencing with the method of sequencing by synthesis, where each tunnel generated millions of raw reads with a sequencing length of 49 bp.

### Annotation of clean tags and data normalization for gene expression level

Raw sequences were transformed into clean tags through removing adaptor sequence, low-quality sequences [tags with unknown sequences (“N”)], empty reads (sequence with only adaptor sequences but no tags), too-long or too-short tags, and tags with a copy number of one (probably a sequencing error). For annotation, all tags were mapped to the reference database (http://www.cassavabase.org, Yang et al., 2011; Prochnik et al., 2012). Clean tags mapped to reference sequences from multiple genes were filtered, and the remaining clean tags were designed as unambiguous tags. For gene expression analysis, the number of unambiguous clean tags for each gene was calculated and then normalized to the number of transcripts per million tags.

### Analysis of DEGs

A rigorous algorithm was developed to identify DEGs among SE, FFEC, and OFEC samples, according to the method described in previous studies (Audic and Claverie, 1997; Liu et al., 2014). The false discovery rate (FDR) was used to determine the threshold of *P*-value in multiple tests and analyses. “FDR ≤ 0.001 and the absolute value of Log_2_ (sample 1/sample 2) ≥ 1” was used as the threshold to judge the significance of gene expression difference (Benjamini and Yekutieli, 2001).

### GO functional enrichment analysis and pathway enrichment analysis

The GO enrichment analysis based on the Gene Ontology database (http://www.geneontology.org) was used to identify the significantly enriched GO terms in DEGs as described in a previous study (Liu et al., 2014). The GO terms with Bonferroni-corrected *P* ≤ 0.05 were significantly enriched in DEGs. The pathway enrichment analysis based on the Kyoto Encyclopedia of Genes and Genomes (http://www.genome.jp/kegg/) was used to identify significantly enriched pathways in DEGs as described in a previous study (Liu et al., 2014). FDR-corrected *Q*-value was used for determining the threshold of *P*-value in multiple tests and analysis (Benjamini and Yekutieli, 2001). Pathways with *Q* ≤ 0.05 were significantly enriched in DEGs.

### Real-time reverse transcription–polymerase chain reaction (qRT-PCR) analysis

To confirm the results of the DEG analyses, the expression of 32 responsive genes was validated by qRT-PCR with RNA samples extracted with a Plant RNA Reagent (Cat.No.12322-02; Invitrogen, Shanghai, China), according to the manufacturer's protocol. The RNA quality was determined by running an agarose gel with ethidium bromide staining. Reverse transcription was performed according to the manufacturer's protocol (Code: TRT-101; TOYOBO, Shanghai, China). Each cDNA sample was diluted two times in sterile ddH_2_O, and 1 μl of this dilution was used as a template for the real-time RT-PCR. Specific primers were designed with Primer-BLAST (http://www.ncbi.nlm.nih.gov/tools/primer-blast) to obtain a *T*_m_ of 60°C and an amplicon length between 70 and 200 bp. The real-time RT-PCR reactions were performed in a 20 μl volume containing 10 μl of 2 × SYBR Green Master Mix (Code: QPK-201, TOYOBO), approximately 50 ng cDNA, and 400 nM of forward and reverse primers in a Bio-Rad CFX96 thermocycler. The amplification conditions were as follows: 95°C for 1 min, followed by 40–50 cycles of 95°C for 15 s, 60°C for 20 s, and 72°C for 20 s. A melting curve (65°C–95°C with a heating rate of 0.05°C/s and a continuous fluorescence measurement) was run after the PCR cycles. Beta-actin was used as the internal control. All the samples were measured in triplicate, and the experiments were performed on three biological replicates. The comparative *C*_*t*_ method was used to calculate the relative gene expression level across the samples. The relative expression level of each gene in one sample (Δ*C*_*t*_) was calculated as follows: *C*_*t*_ (target gene)–*C*_*t*_ (beta-actin). The relative expression of each gene in two different samples (ΔΔ*C*_*t*_) was calculated as follows: Δ*C*_*t*_ (sample 1)–Δ*C*_*t*_ (sample 2).

### HPLC estimation of global methylation rates

A modified cetyltrimethylammonium bromide (CTAB) method was used for genomic DNA extraction (Soni and Murray, 1994). Purified DNA (100 μl, about 3 μg) was hydrolyzed into nucleosides for 1 h at 95°C by adding 50 μl 70% perchloric acid, and the pH was adjusted to 3–5 with 5 M KOH (about 100 μl); then the samples were centrifuged at 12,000 rpm for 10 min. The supernatant was transferred to a new tube and analyzed using an HPLC 1260 system. The HPLC column (ZORBAX carbohydrate column; 4.6 × 150 mm, 5 μm) of Agilent technologies with a UV detector was used. The detection wavelength was fixed to 285 nm. The mobile phase consisted of 75% acetonitrile with a flow rate of 0.8 ml/min, and the temperature of the column was maintained at 35°C. Cytosine (C3506, Sigma, MO, USA) and 5-methylcytosine hydrochloride (M6751, Sigma) were used as standards. The cytosine and 5-methylcytosine were identified based on the retention time of the standards, and sample concentrations were calculated from the external standard curve. The global methylation rate (GMR) was calculated using the following formula: GMR = (5mC)/[(C) + (5mC)] × 100%. All analyses were repeated three times, and the mean ± standard error (SE) was calculated.

### Flow cytometry analysis

The harvested samples of 0.3 g were gently chopped with a razor blade in a petri dish containing 1.5 ml modified Galbraith's extraction buffer (Galbraith et al., 1983) supplemented with 5 mM sodium metabisulfite and 0.5% mercaptoethanol. The chopped samples were then filtered through a 40 μm mesh, then a 7.5 μl DAPI buffer (final concentration 5 μg/ml) was added to it and kept in dark for 5 min. The isolated nuclei were analyzed at a concentration of 150,000 cells per sample using a MoFlo XDP flow cytometer. Obtained histograms were evaluated using the Summit 5.2 software (Beckman Coulter, CA, USA, http://www.beckmancoulter.com). Results from two biological replicates, each with duplicated samples were analyzed. The leaves from TMS60444 *in vitro* were treated as control with the fluorescence peak at channel position 1.7 × 10^4^ (FL6-Log_Height). The S-phase fraction (SPF) was calculated using the following formula: SPF = S/(G_0_/G_1_+ S + G_2_/M) × 100%. The proliferation index (PI) was calculated using the following formula: PI = (S + G_2_/M)/(G_0_/G_1_+ S + G_2_/M) × 100%.

## Results

### Cytological and physiological changes of SEs, FFEC, and OFEC

**Figure 1:**
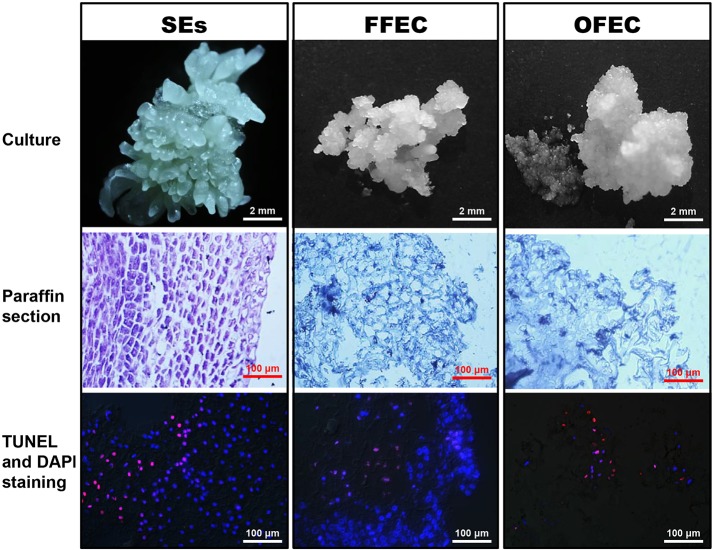
**SEs, FFEC, and OFEC of cassava and observation of their TUNEL- and DAPI-stained paraffin sections**.

The SE clusters usually contained mixed types of SEs with global, heart, torpedo, and cotyledon stages, which were ready for further multiplication on a cassava induction medium (CIM) containing 50 μM picloram or to be used for FFEC induction on a GD medium under continuous darkness (Figure 1). The SE cells were well-arranged with dense cytoplasm and absence of DNA fragmentation as indicated by TUNEL- and DAPI-stained paraffin sections. The FFEC was a light yellow, highly friable callus with numerous spherical embryogenic units and sparse cytoplasm with less well-arranged nuclei. The OFEC was pale yellow and less well-organized having sponge-like cell structure with few detectable nuclei (Figure 1). OFEC showed lower plant regeneration capacity and usually gave abnormal phenotype, such as dwarf, yellow, and/or slim, or twisted leaves (data not shown).

**Figure 2:**
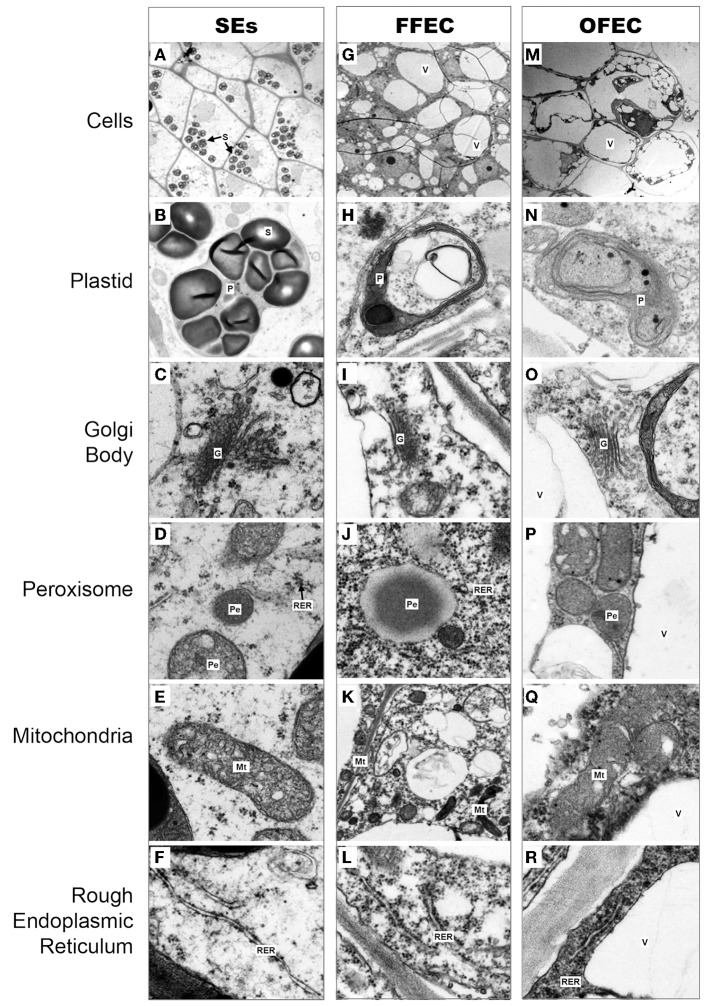
**Cytological observations of cells in SEs (A–F), FFEC (G–L), and OFEC (M–R) of cassava by TEM**. G, Golgi body; Mt, mitochondria; P, plastid; Pe, peroxisome; RER, rough endoplasmic reticulum; S, starch granule; V, vacuole.

Under a transmission electron microscope, the isodiametric SE cells were detected and their cell cytoplasm was distributed uniformly with a clear cell wall boundary (Figure 2A). Both FFEC and OFEC cells showed irregular shapes with very thin cell walls and contained large vacuoles, but the FFEC cells showed denser cytoplasm compared with the OFEC cells. Two types of cells were identified in FFEC, one with small vacuolar vesicles and another with a larger vacuole as in the OFEC cells. Some of the OFEC cells contained debris and were less organized (Figures 2G,M). A number of starch granules were observed inside the SE cells with several starch granules in one plastid (Figure 2B). No starch granule was found in the FFEC and OFEC cells (Figures 2H,N). When checking the subcellular organelles, the SEs detected regular Golgi body, mitochondria, and rough endoplasmic reticulum (RER). These organelles of FFEC and OFEC had irregular shapes and were consistent with higher dense RER and more binding ribosomes (Figures 2C–E,I–K,O–Q). Free ribosomes became enriched in FFEC and OFEC, which indicated active protein synthesis (Figures 2F,L,R).

**Figure 3:**
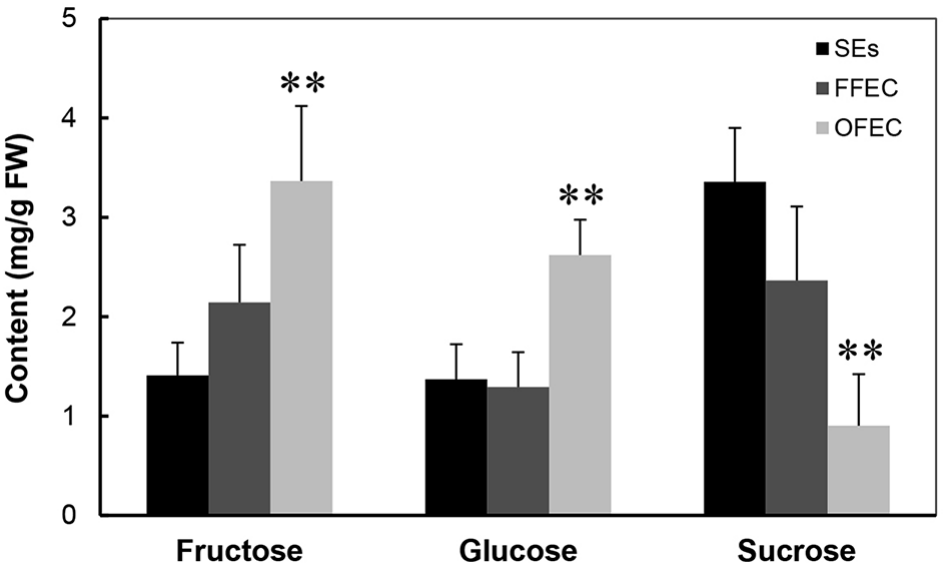
**Contents of fructose, glucose, and sucrose in SEs, FFEC, and OFEC**. The double asterisks indicate a statistically significant difference at *P* < 0.05 by Student's *t-*test. The mean values are calculated from three biological replicates, and the error bars represent the standard error of the mean (SEM).

The analysis of sugars by HPLC indicated that the contents of fructose and glucose increased significantly but the sucrose content decreased dramatically in OFEC (Figure 3), indicating that the status of sucrose homeostasis is changed during the long-term subculturing process. No significant difference of fructose and glucose contents was found between SEs and FFEC.

### Solexa sequencing and annotation analysis revealed dynamic gene expression among SEs, FFEC and OFEC

Using the Solexa sequencing technology, the genome-wide gene expression change in SEs, FFEC, and OFEC was analyzed. The copy number distribution of the clean tags and the saturation evaluation of different expressions validated the sequencing quality. The distribution of total tags and distinct tags over different tag abundance categories showed similar patterns for three DEG libraries, suggesting there was no bias in the construction of the libraries from SEs, FFEC, and OFEC (data not shown). To explore the DEGs in the three samples, three DEG libraries were mapped to the reference database of cassava-expressed sequence tags, which generated about 4.90 million raw tags for each sample (Supplementary Table 1). After the removal of the low-quality reads, the total number of tags per library ranged from 4.67 to 4.70 million and the number of distinct tags ranged from 173,690 to 203,119. About 34.81–46.33% distinct tags in all clean tags were mapped to reference genes, and 19,800, 19,840, and 19,366 unambiguous tag-mapped genes were identified in SE, FFEC, and OFEC samples, respectively (Supplementary Table 1).

**Figure 4:**
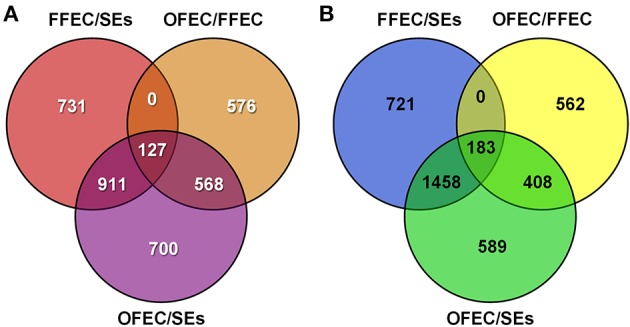
**The Venn diagram depicts the (A) upregulated and (B) downregulated genes in paired comparisons**. FFEC/SEs, fresh friable embryogenic callus/somatic embryos; OFEC/FFEC, old friable embryogenic callus/fresh friable embryogenic callus; OFEC/SEs, old friable embryogenic callus/somatic embryos.

Using the criteria of FDR < 0.001 and a two-fold change in expression, 6871 DEGs were identified in the three samples (Figure 4). Among the DEGs, 2306, 1769, and 1271 genes were upregulated and 2683, 2362, and 1153 were downregulated by analyzing OFEC/SEs, FFEC/SEs, and OFEC/FFEC, respectively. In three comparisons, 127 and 183 genes were upregulated and downregulated, respectively (Figure 4). The DEGs were more in OFEC/SEs and FFEC/SEs than in OFEC/FFEC, implying that FFEC, and OFEC were closer in genetic status compared with SEs, which was consistent with the biological process as well as cytological observations.

To validate the Solexa sequencing data, the qRT-PCR analysis was performed on 18 selected DEGs belonging to different functional categories or pathways (Table 1). There were 94.44, 72.22, and 100.00% consistency between the results measured by real-time RT-PCR and RNA-seq among FFEC/SEs, OFEC/FFEC, and OFEC/SEs, respectively.

**Table 1:** **Validation of selected gene expression by real-time RT-PCR**.

**Genes**	**Description**	**Solexa**			**qRT-PCR**		
		**FFEC/SEs**	**OFEC/FFEC**	**OFEC/SEs**	**FFEC SEs**	**OFEC/FFEC**	**OFEC/SEs**
Cassava4.1_000881m	Histidine kinase cytokinin receptor, Mrna (CRE)	−3.53	1.19	−2.34	−3.67	2.76	−0.91
Cassava4.1_012131m	NAD^+^	4.14	−0.58	3.56	4.94	**0.87**	5.81
Cassava4.1_004367m	Acetyl-CoA synthetase	3.32	−0.11	3.21	0.97	**0.90**	1.87
Cassava4.1_019292m	Auxin-responsive protein IAA	−10.63	−	−10.63	−1.81	−**0.32**	−2.13
Cassava4.1_003815m	Auxin-responsive GH3 gene family	−5.74	−1.41	−7.16	−1.46	−0.86	−2.32
Cassava4.1_019233m	SAUR family protein	−10.42	7.71	−2.71	−2.47	1.74	−0.73
Cassava4.1_004821m	Pectinesterase-3 precursor	−8.12	5.39	−2.73	−4.21	2.32	−1.89
Cassava4.1_006815m	Predicted: similar to TUBG1 (gamma-tubulin)	−8.23	7.85	−0.38	−2.62	2.40	−0.22
Cassava4.1_001933m	Kinesin	−7.00	8.40	1.40	−2.23	2.25	0.02
Cassava4.1_008560m	Cell division protein FtsZ	−0.58	1.39	0.80	**0.38**	1.02	1.40
Cassava4.1_010144m	Cyclin A	−6.74	8.40	1.66	−2.27	5.14	2.87
Cassava4.1_005111m	GLN phosphoribosyl pyrophosphate amidotransferase 2	1.19	0.31	1.51	1.42	0.77	2.18
Cassava4.1_009073m	Phosphoribosyltransferase family protein	1.58	0.24	1.82	0.40	0.69	1.09
Cassava4.1_010597m	Glutamine synthase clone R1	2.28	−0.01	2.27	2.59	**0.40**	2.99
Cassava4.1_006179m	Glutamate decarboxylase	−3.23	1.50	−1.73	−4.91	2.84	−2.07
Cassava4.1_004336m	Phosphoglucomutase/phosphomannomutase family protein	1.32	0.12	1.43	0.47	0.77	1.24
Cassava4.1_006657m	UDP-glucose 6-dehydrogenase family protein	−1.90	−0.50	−2.40	−1.81	**0.87**	−0.93
Cassava4.1_031387m	Xylan 1,4-beta-xylosidase	−8.23	5.39	−2.84	−2.83	1.98	−0.85

*All data are shown in log_2_ratio, and the positive and negative values of log_2_ ratio are either up- or down-regulated genes in the three paired comparisons. No significant fold changes are indicated as “−.” Values of real-time RT-PCR analysis shown in bold font indicate inconsistent data compared with Solexa analysis*.

### GO clustering of degs and clustering of significant pathways involved

**Figure 5:**
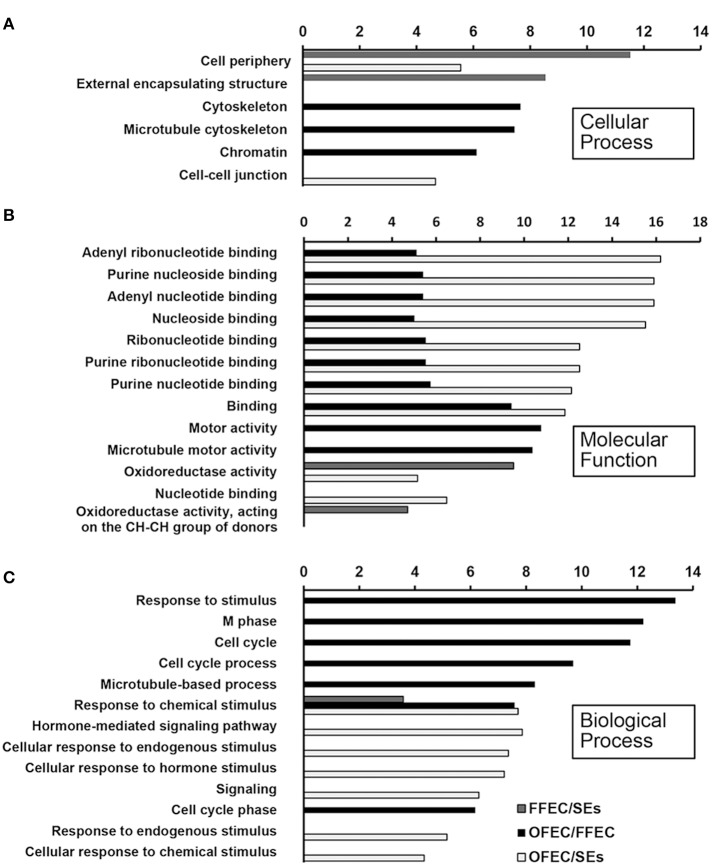
**Significantly enriched GO terms of “cellular process” (A), “molecular function” (B), and “biological process” (C) by comparing SEs, FFEC, and OFEC (*P* ≤ 0.05)**. The “*y*” axis represented enriched GO terms among three samples. The “*x*” axis represented the *P*-value (−log_2_ *P*).

**Figure 6:**
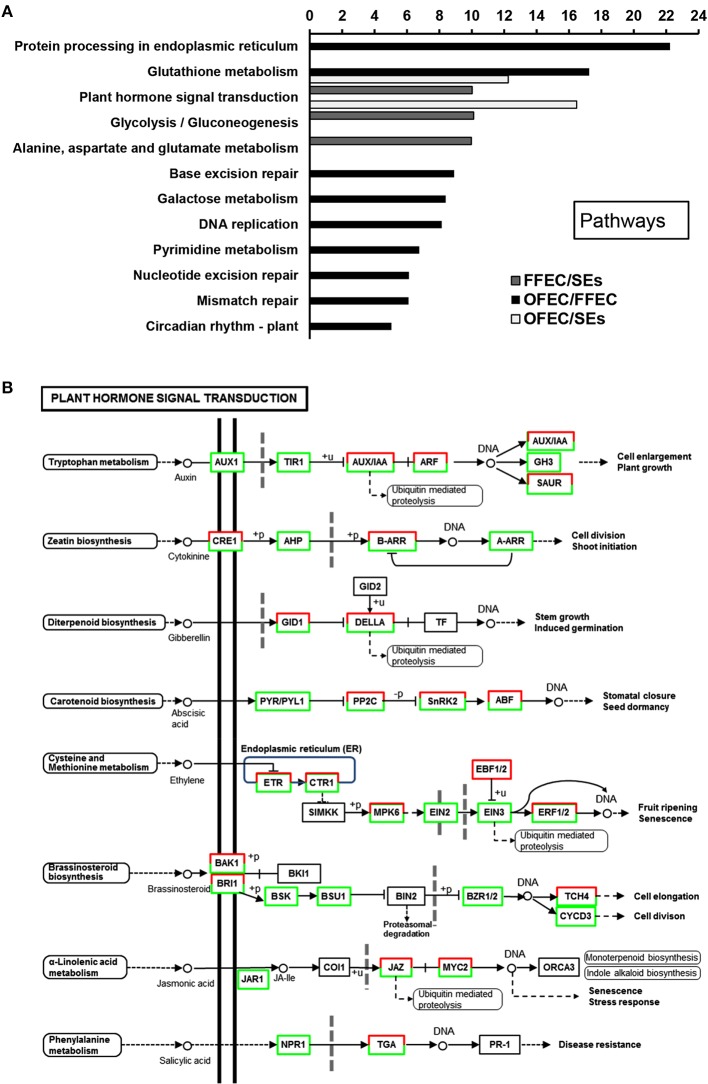
**Significantly enriched pathways (A) and changes of plant hormone signal transduction (B) by comparing SEs, FFEC, and OFEC (*P* < 0.05)**. The “*y*” axis represented enriched pathways among the three samples, and the “*x*” axis represented the *P*-value (−log_2_ *P*).

The GO terms of “cellular process,” “molecular function,” and “biological process” were significantly enriched among SEs, FFEC, and OFEC (Figure 5). Of them, the categories including “cell periphery,” “external encapsulating structure,” “oxidoreductase activity,” and “response to chemical stimulus” were significantly regulated in FFEC (Supplementary Table 2). In OFEC, the main categories contained terms for “cell cycle related,” “cytoskeleton related,” “chromatin,” and “nucleotide related” (Figure 5). According to the threshold of *P* < 0.05 and *Q* < 0.05, 14 significant pathways were identified (Figure 6A). In FFEC, the main pathways included were “plant hormone signal transduction” (ko04075), “glycolysis/gluconeogenesis” (ko00010), and “alanine, aspartate, and glutamate metabolism” (ko00250). Different pathways involved in OFEC are “base excision repair” (ko03410), “DNA replication” (ko03030), “nucleotide excision repair” (ko3420), and “mismatch repair” (ko3430), all of which were related to the DNA synthesis and repair process (Figure 6A).

### Changes during the FEC formation process (FFEC/SEs)

The “plant hormone signal transduction” (ko04075) pathway was significantly enriched (Figure 6B); in this pathway, a number of growth-associated processes were activated, including tryptophan metabolism (auxin), zeatin biosynthesis (cytokinine), diterpenoid biosynthesis (gibberellin), cysteine, and methionine metabolism (ethylene), and brassinosteroid biosynthesis (brassinosteroid) (Supplementary Table 3). These processes played a crucial role in the FEC formation process for regulating elongation and cell division. In the tryptophan metabolism (auxin) pathway, most of the genes involved in this metabolism were downregulated except the auxin-induced protein X10A and the auxin-responsive protein IAA16.

In the diterpenoid biosynthesis (gibberellin) process, the genes of GA including cyclin D, glucan endo-1,3-beta-glucosidase precursor, protein kinase, and protein phosphatase 2c play functions in the cell reconstruction of structure and endomaterials. In this study, gibberellin receptor GID1, GRAS family transcription factor (TF), and ubiquitin-protein ligase were upregulated, but DELLA protein was downregulated (Supplementary Table 3). The DELLA protein was degraded by ubiquitin-mediated gibberellin receptor GID1 that combined with GA; therefore, the genes related with GA were induced, which could lead to cell reconstruction.

In the GO terms of “cellular process,” the largest and second categories of “cell periphery” and “external encapsulating structure,” accounting for 7.80 and 6.70% of the total GOs, respectively, might be the most important components related to the FEC formation. Since all genes in the category of “external encapsulating structure” were overlapping with genes in the GO terms of “cell periphery,” the two categories were combined for analysis and discussion (Supplementary Table 2). Among the 57 genes significantly changed at FFEC/SEs, 42 genes were downregulated and 15 genes upregulated. Many genes that loosen the cell wall structure were detected, including alpha-expansin 11 precursor, xyloglucan endotransglucosylase/hydrolase protein 22 precursor (XTH), pectin acetylesterase, and so on. Many genes involved in the polysaccharide hydrolase of cell wall were observed, such as alpha-galactosidase, alpha-glucosidase, beta-galactosidase, glucan endo-1,3-beta-glucosidase precursor, xylan 1,4-beta-xylosidase, hydrolyzing *O*-glycosyl compounds, periplasmic beta-glucosidase precursor, acid beta-fructofuranosidase precursor, triosephosphate isomerase, and so on. For example, pectinesterase precursors and glucan endo-1,3-beta-glucosidase precursors were downregulated dramatically, and pectin acetylesterase (cassava4.1_ 032451m|pacid:17979110) and alpha-galactosidase/alpha-*N*-acetylgalactosaminidase (cassava4.1_011258m|pacid:17973258) were upregulated.

The genes of protein hydrolysis, including 26S protease regulatory subunit S10b, aspartic proteinase nepenthesin-1 precursor, and other genes were all significantly downregulated. The factors responsible for external stimulus betaine-aldehyde dehydrogenase, multicopper oxidase, and leucine-rich repeat in FFEC had also downregulated expression compared with that in SEs. The genes, for example, similar to TUBG1 (gamma-tubulin), involved in cytoskeleton construction were downregulated while fimbrin and tubulin alpha-8 chain were significantly upregulated in FECs compared to SEs.

Furthermore, other genes containing a wide range of biological functions were detected, including protein kinase (e.g., serine–threonine protein kinase), heat shock proteins (e.g., heat shock protein), protein synthesis (e.g., 40S ribosomal protein S27), protein folding (e.g., calnexin), lipid metabolism (e.g., acid phosphatase one precursor), nucleotide metabolism (e.g., amidophosphoribosyltransferase), and auxin-responsive protein (e.g., GAST1 protein precursor). Moreover, the transcription factors (TFs) differentially expressed during the culture process were also monitored (Supplementary Table 6). Significant upregulation of TFs (e.g., AP2/ERF TF, MADS-box TF 2, R2R3-MYB TF, BIM1 TF, and WRKY TF) was detected in FFEC/SEs (log_2_ratio >5) but more down-regulated TFs were also found during the process (log_2_ratio < −5). Interestingly, most of these TFs down-regulated in FFEC/SEs were up-regulation in OFEC/FFEC, indicating the regulation of FEC induction is different from the FEC subculture process.

### Rapid cell cycle and repair mechanism during the FEC continuous subculture process (OFEC/FFEC)

Many significantly expressed pathways, including “base excision repair,” “DNA replication,” “nucleotide excision repair,” and “match repair,” involved in DNA replication or repair, were found during the FEC continuous subculture process (OFEC/FFEC) (Supplementary Table 4). In these pathways, many important genes related to the DNA damage–induction repair process, such as replication factor C (RFC), proliferating cell nuclear antigen (PCNA), and replication protein A (RPA), were also significantly upregulated.

**Figure 7:**
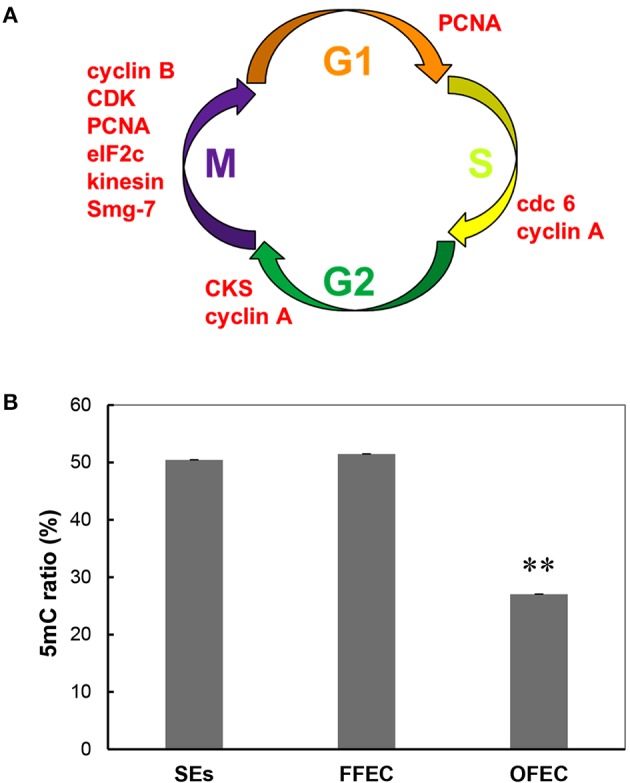
**Upregulated genes related to cell cycle (A) and genomic DNA methylation ratio (B) during the culture process from SEs to OFEC**. The mean values are calculated from three biological replicates, the error bars represent the standard error of the mean (SEM). The two asterisks indicates a statistically significant difference at *P* < 0.01.

In the GO terms of “molecular function,” the “cell cycle–related terms” were identified. Since many genes in the category of “cell cycle” overlapped with genes in the GO terms of “cell cycle process” and “M phase,” these categories were combined for analysis and discussion. All genes in this term, including cyclin B, cdc6, kinesin, CDK, smg-7, cyclin A, and so on, were significantly upregulated at OFEC/FFEC, except for cop9 complex subunit (Figure 7A, Supplementary Table 5).

Among the significant upregulated TF (log_2_ratio > 5) in OFEC/FFEC, one third of TFs showed upregulation in FFEC/SEs, including ethylene-responsive TF, R2R3-MYB TF, MADS-box TF 3 and WRKY TFs; The rest of them were considerably down-regulated in FFEC/SEs (log_2_ratio < −5) (Supplementary Table 6). Some TFs might be related to the cell cycle progress, such as TF E2F and TF DP-1. Overall, the changes of TF expression showed different scenarios between OFEC/FFEC and FFEC/SEs.

### Methylation levels reduced in OFEC

DNA methylation dysfunctions can lead to developmental abnormalities in animals and plants (Finnegan et al., 2000). To evaluate the methylation levels in SEs, FEC, and OFEC and possible causes in high somaclonal variation of regenerated cassava plants from OFEC, the global methylation ratios were evaluated by the HPLC analysis. The ratio of methylation had no significant changes between SEs and FFEC, both were about 50%. However, the ratio of methylation in OFEC decreased significantly, which was almost 27% (Figure 7B).

### Unchanged ploidy level in SEs, FFEC, and OFEC

**Figure 8:**
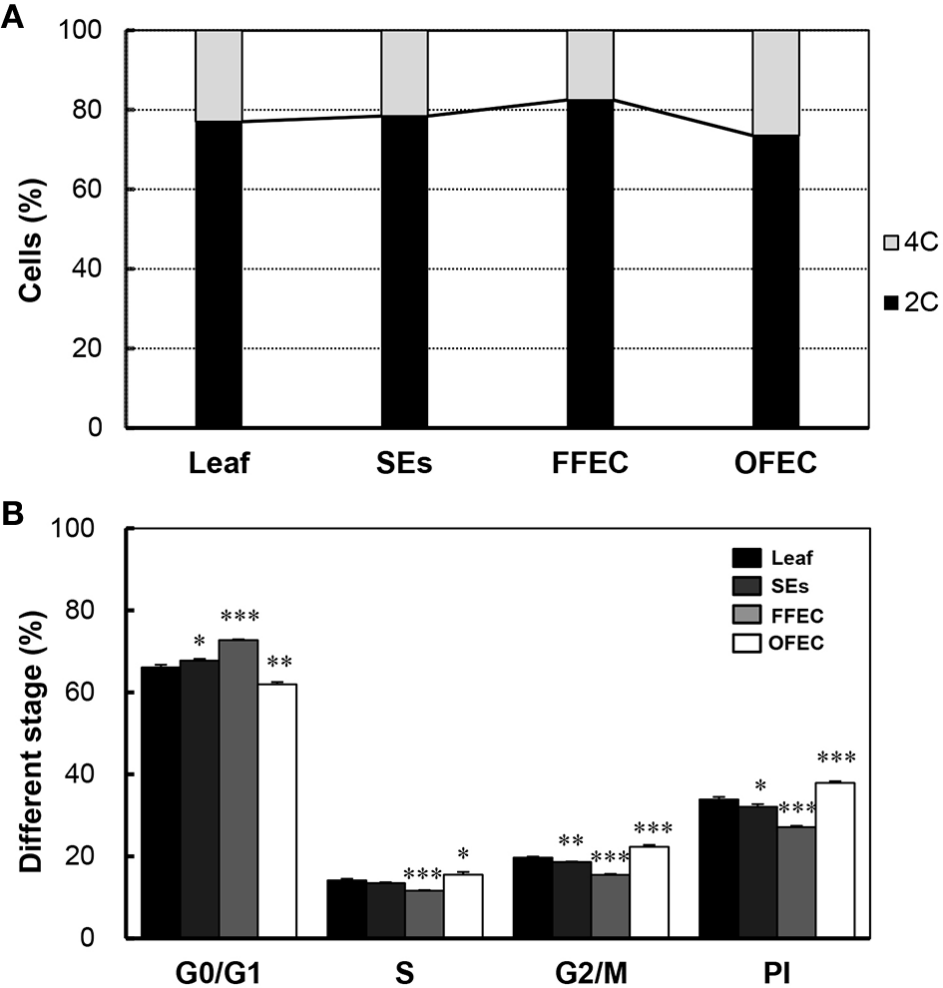
**Flow cytometric analysis of leaf, SEs, FFEC, and OFEC**. **(A)** Predominant G1/G0 peak (channel 1.7 × 10^4^) of the diploid (2*n* = 2*x* = 36) and their relative ratios of 2C and 4C cells. **(B)** The percentage of cells in G0/G1, S, and G2/M phases. PI indicates the proliferation index, PI = (S + G2/M)/(G0/G1 + S + G2/M) × 100%. The single, double, and triple asterisks indicate a statistically significant difference at *P* < 0.05, *P* < 0.01, and *P* < 0.001, respectively. The mean values are calculated from three biological replicates, and the error bars represent the standard error of the mean (SEM).

Flow cytometry analysis was performed to examine the nuclear ploidy during FEC formation and continuous culturing process. The DNA contents of SEs, FFEC, and OFEC analyzed by flow cytometry were similar to that of the leaves, which indicated that the ploidy of SEs, FFEC, and OFEC had no difference (Supplementary Figures 1A–D). Nevertheless, the OFEC cells contained more 4C cells (26.5% of total 4C and 2C cells) compared with SE and FFEC cells. The FFEC cells only had 17.6% 4C cells (Figure 8A). The OFEC cells contained the highest ratios of SPF and PI, 15.57, and 37.92%, respectively, compared to the SE and FFEC cells (Figure 8B). Since most of cell cycle-related gene were also up-regulated (Figure 7A, Supplementary Table 5), these data indicate the OFEC cells possessed stronger proliferation ability during the subculture process.

## Discussion

Callus induction is a complicated biological process of cell differentiation and dedifferentiation regulated by genetic and epigenetic mechanisms (Ikeuchi et al., 2013). Despite the importance of FEC as a key tool for cassava genetic engineering (Liu et al., 2011), less studies have been made to understand the mechanisms underlying the FEC induction and long-term subculture. In this study, transcriptomic, structural, and physiological changes were analyzed during the FEC induction and subculture process. The activation of hormone signal transduction pathway played a key role in inducing the expression of genes participating in cell reconstruction for the transformation from SE cells to FFEC. During the FEC subculture process, the cell cycles were accelerated by upregulating the cell cycle–related genes accompanying the dramatic reduction of the methylation level and changed expression of TFs. The gene repair mechanism was also enhanced. The acceleration of cell division in FEC potentially resulted in more somaclonal variations during FEC subculture. Hence, the study increased the understanding of genetic and physiological changes during the FEC formation and subculture, providing cues to develop a robust protocol for farmer-preferred or recalcitrant cultivars.

The transition from SEs to FFEC is the key limiting factor in the cassava genetic transformation system. Structural changes, mainly in the cell wall, were proved to be critical for callus formation (Ikeuchi et al., 2013), and loosening of the cell wall was noticed in the isodiametric conjunction of SEs cultured on a GD medium for 2 weeks during the FEC induction process. Much thinner cell wall existed in FFEC and OFEC (Figure 2). The GO terms of the largest and second categories of “cell periphery” and “external encapsulating structure” were enriched in the “cellular component,” indicating that FEC formation was associated with cell reconstruction in SEs. Totally 15 genes were significantly upregulated and 42 genes were downregulated during the transition process from SEs to FFEC, including the genes of cell wall formation (Supplementary Table 2). For example, XTHs that mediate cleavage and rejoining of the β (1–4)-xyloglucans of the primary cell wall seem to achieve regulated wall loosening during turgor-driven expansion by rearranging load-bearing xyloglucan cross-links between cellulose microfibrils (Jan et al., 2004; Saladie et al., 2006). Pectin acetylesterase modulates the precise status of pectin acetylation to affect the remodeling and physiochemical properties of the cell wall's polysaccharides, thereby affecting cell extensibility (Gou et al., 2012). Pectinesterase could catalyze the de-esterification of pectin into pectate and methanol, and stiff the cell wall by producing blocks of unesterified carboxyl groups that can interact with calcium ions forming a pectate gel (Micheli, 2001). The transcription of all these genes decreased significantly in FFEC/SEs. The genes such as xylan 1,4-beta-xylosidase, which catalyzed the breakage of glycosidic bond to hydrolyze cell wall into small polymer (Dilokpimol et al., 2011), were upregulated. Indeed, the mechanical damage such as wounding organized embryogenic structures to encourage FEC production had been reported by a previous study (Taylor et al., 2012). These genes might play a crucial role in the FEC induction process through participating in cell reconstruction.

The hormone signal transduction and cell cycle–related categories were enriched at OFEC/SEs and OFEC/FFEC, respectively. The components in the “plant hormone signal transduction” (ko04075) pathway were activated (Figure 6B), including tryptophan metabolism (auxin), zeatin biosynthesis (cytokinine), diterpenoid biosynthesis (gibberellin), cysteine, and methionine metabolism (ethylene), and brassinosteroid biosynthesis (brassinosteroid), which was consistent with the results of previous research. The pathways of plant hormone signal transduction were enriched during the initiation of embryogenic tissues (Elhiti et al., 2013; Li et al., 2014). But the previous studies indicated that hormonal signaling networks could establish functional links between the regulatory mechanisms of the cell cycle and genes involved in callus formation (Pischke et al., 2006), and the callus-forming capacity in hypocotyls with age may work in concert to altered cytokinin signaling components, cell cycle regulation, and epigenetics (Chen et al., 2012). Dramatic changes of TF expression during FEC subculturing were detected and some TFs are hormone responsive-related (Supplementary Table 6). These data support that the hormone response changed greatly over the long-term subculture, leading to dramatic changes in cell status genetically and epigenetically.

Starch granules were only discovered in plastids of SEs (Figure 2B), indicating SEs had different sucrose and starch metabolism from FFEC and OFEC. Increase of fructose and glucose and decrease of sucrose in OFEC indicated its vigorous sugar metabolism during the FEC subculture process. The OFEC cells might consume more energy in the process of cell proliferation due to its rapid cell division. In Valencia sweet orange, histological study of non-embryogenic, and embryogenic callus showed that starch accumulation in amyloplasts of embryogenic cells was obviously increased (Ge et al., 2012). Therefore, it might be necessary to gradually increase the sucrose content from normal 3 to 6% during FEC subculture to maintain the embryogenic nature of FEC. In fact, the embryogenic suspension established by culturing FEC in the liquid SH medium contains high sucrose content of 6% (Zhang and Gruissem, 2004). Additionally, many pathways involving protein processing in ER; glutathione metabolism; and alanine, aspartate, and glutamate metabolism were also changed. Indeed, supplementing additional amino acids, for example, tyrosine, in cassava could enhance FEC induction in a genotype-dependent manner (Nyaboga et al., 2013).

Severe somaclonal variations in regenerated plants were noticed when using cassava FEC system, such as dwarf, yellow, and/or slim, or twisted leaves, even white plants (Raemakers et al., 2001; Taylor et al., 2004). The GO terms of “molecular function” at OFEC/FFEC partly explained the appearance of somaclonal variation. Most genes were upregulated except for cop9 complex subunit, including cyclin A, cyclin B, Cdc6, CDK, kinesin, PCNA, eukaryotic translation initiation factor 2c (eIF 2C), and so on. Apparently, the cell cycles were fastened during the continuous subculture of FEC, which is further manifested by the flow cytometry analysis in which the OFEC cells contained the highest ratios of SPF and PI (Figure 8B). No change of ploidy levels of FEC was observed during subculture. Furthermore, four pathways related with DNA repair and mutation were significantly enriched at OFEC/FFEC, including “base excision repair,” “DNA replication,” “nucleotide excision repair,” and “match repair.” The genes participating in DNA repair and replication were significantly upregulated, including RFC, PCNA, and RPA (Singh et al., 2010). Many TFs were identified and showed different expression scenarios among FFEC/SEs, OFEC/FFEC and OFEC/SEs (Supplementary Table 6). Therefore, mutagenesis during the continuous FEC subculture process can occur with the increasing DNA damage and repair.

Epigenetic regulation plays an important role in callus formation by affecting gene expression via chromatin modification including DNA methylation and histone modification (Ikeuchi et al., 2013). The DNA methylation ratio of OFEC almost reduced two-fold compared with the SE and FFEC cells, indicating many genes had different expression profiles in the OFEC cells, including the genes with cell cycles and DNA replication, DNA repair, and unknown function, which might partly lead to the somaclonal variation of the regenerated plants (Rival et al., 2013). Plants with decreased methylation displayed a number of phenotypic and developmental abnormalities, including reduced apical dominance, smaller plant size, altered leaf size, and shape, decreased fertility, and altered flowering time, and so on (Finnegan et al., 2000).

## Conclusions

In summary, the study presented a genome-wide gene expression profiling related to FEC formation and subculture process of TMS60444 cultivar by the use of RNA-seq. The important event for FEC induction for SEs is the cell reconstruction with the loosening cell wall. Changing the basic medium components, especially the micronutrients, might facilitate the FEC induction. The increase of sucrose content also can delay the occurrence of somaclonal variation during FEC subculture. Certain amino acids might be helpful for FEC induction under the genotype-dependent manner. The rapid cell division and epigenetic regulation by reduced DNA methylation are enhanced during the FEC subculture process and leads to vulnerability of somaclonal variation. This study would shed light for the FEC induction from recalcitrant cultivars and improve the efficiency of FEC-based genetic transformation of cassava.

## Author contributions

QM carried out the cassava SE and FEC production, molecular, and physiological analysis, and wrote the manuscript. WZ conducted the methylation analysis and sugar measurement. PZ was responsible for the overall concept, experimental design, data analysis, and writing this manuscript. All authors read and approved the manuscript.

### Conflict of interest statement

The authors declare that the research was conducted in the absence of any commercial or financial relationships that could be construed as a potential conflict of interest.

## Abbreviations

DEG: differentially expressed gene
FEC: friable embryogenic callus
FFEC: fresh FEC
GO: Gene Ontology
HPLC: high-performance liquid chromatography
OFEC: old FEC
qRT-PCR: real-time reverse transcription–polymerase chain reaction
RNA-seq: RNA sequencing
SE: somatic embryo
TEM: transmission electron microscopy
TF: transcription factor.
