# Supplementary material for: Transition from somatic embryo to friable embryogenic callus in cassava: dynamic changes in cellular structure, physiological status, and gene expression profiles
Source: Front Plant Sci. 2015 Oct 6;6:824. doi: 10.3389/fpls.2015.00824 (PMC4594424; doi:10.3389/fpls.2015.00824)
Supplement: Supplementary file 1 [file Table1.DOCX]

| **Supplementary Table 1.**  Statistics of DEGs sequencing from SEs, FFEC and OFEC | | | | |
| --- | --- | --- | --- | --- |
| Category | Parameter | Value of SEs | Value of FFEC | Value of OFEC |
| Raw tag | Total No. of tags | 4,883,525 | 4,949,151 | 4,961,894 |
|  | No. of distinct tags | 385,206 | 456,677 | 382,234 |
| Clean tag | Total No. of tags | 4,672,920 | 4,695,412 | 4,753,155 |
|  | No. of distinct tags | 174,810 | 203,119 | 173,690 |
| All tags mapping to gene | No. of distinct tags | 78,272 | 70,704 | 80,467 |
|  | Distinct tag % of clean tags | 44.78% | 34.81% | 46.33% |
| All tag-mapped genes | No. of genes | 19,882 | 19,904 | 19,435 |
|  | % of ref. genes | 58.22% | 58.28% | 56.91% |
| Unambiguous tag-mapped genes | No. of genes | 19,800 | 19,840 | 19,366 |
|  | % of ref. genes | 57.98% | 58.09% | 56.71% |
