# Supplementary material for: Transition from somatic embryo to friable embryogenic callus in cassava: dynamic changes in cellular structure, physiological status, and gene expression profiles
Source: Front Plant Sci. 2015 Oct 6;6:824. doi: 10.3389/fpls.2015.00824 (PMC4594424; doi:10.3389/fpls.2015.00824)
Supplement: Supplementary file 2 [file Table2.DOCX]

| **Supplementary Table 2.** DEGs involved in the process of ‘Cell periphery’ and ‘External encapsulating structure’ | | | | | |
| --- | --- | --- | --- | --- | --- |
| Genes | FFEC  /SEs | OFEC  /FFEC | OFEC  /SEs | Description | Categories |
| *Cell wall formation* | | | | | |
| cassava4.1_014513m\|pacid:17960426 | -3.31 | -4.11 | -7.42 | Alpha-expansin 11 precursor | cell periphery |
| cassava4.1_014262m\|pacid:17964017 | -1.57 | -1.33 | -2.90 | Beta-expansin 1a precursor | external encapsualting structure/cell periphery |
| cassava4.1_013286m\|pacid:17993141 | -3.09 | 2.26 | -0.83 | Xyloglucan endotransglucosylase/hydrolase protein 22 precursor | external encapsualting structure/cell periphery |
| cassava4.1_011585m\|pacid:17969357 | 1.60 | 0.91 | 2.51 | Xyloglucan endotransglucosylase/hydrolase protein 9 precursor | external encapsualting structure/cell periphery |
| cassava4.1_013014m\|pacid:17977026 | -4.35 | -5.43 | -9.78 | Xyloglucan endotransglucosylase/hydrolase protein A precursor | external encapsualting structure/cell periphery |
| cassava4.1_009152m\|pacid:17981157 | -2.97 | -0.18 | -3.15 | Pectin acetylesterase | external encapsualting structure/cell periphery |
| cassava4.1_024915m\|pacid:17971859 | 1.19 | -0.58 | 0.61 | Pectin acetylesterase | external encapsualting structure/cell periphery |
| cassava4.1_032451m\|pacid:17979110 | 8.11 | 1.64 | 9.75 | Pectin acetylesterase | external encapsualting structure/cell periphery |
| cassava4.1_005186m\|pacid:17992773 | -4.35 | -7.00 | -11.35 | Pectinesterase PPE8B precursor | external encapsualting structure/cell periphery |
| cassava4.1_026531m\|pacid:17993327 | -8.12 | - | -8.12 | Pectinesterase-2 precursor | external encapsualting structure/cell periphery |
| cassava4.1_023656m\|pacid:17989035 | -2.20 | 0.98 | -1.22 | Pectinesterase-2 precursor | external encapsualting structure/cell periphery |
| cassava4.1_004821m\|pacid:17981465 | -8.12 | 5.39 | -2.73 | Pectinesterase-3 precursor | external encapsualting structure/cell periphery |
| cassava4.1_004226m\|pacid:17970175 | -3.57 | -1.53 | -5.10 | Pectinesterase-3 precursor | external encapsualting structure/cell periphery |
| cassava4.1_006449m\|pacid:17972212 | -3.11 | -0.37 | -3.48 | Cel9B | external encapsualting structure/cell periphery |
| cassava4.1_023135m\|pacid:17991879 | -8.94 | - | -8.94 | Alpha-galactosidase/alpha-N-acetylgalactosaminidase | external encapsualting structure/cell periphery |
| cassava4.1_011258m\|pacid:17973258 | 3.89 | -0.21 | 3.68 | Alpha-galactosidase/alpha-N-acetylgalactosaminidase | external encapsualting structure/cell periphery |
| cassava4.1_001205m\|pacid:17962849 | -1.44 | 0.89 | -0.55 | Alpha-glucosidase | external encapsualting structure/cell periphery |
| cassava4.1_001635m\|pacid:17982425 | 2.54 | -0.69 | 1.85 | Beta-galactosidase | external encapsualting structure/cell periphery |
| cassava4.1_001733m\|pacid:17990759 | 3.87 | -2.98 | 0.89 | Beta-galactosidase | external encapsualting structure/cell periphery |
| cassava4.1_005968m\|pacid:17964195 | -2.77 | 1.44 | -1.33 | Glucan endo-1,3-beta-glucosidase precursor | external encapsualting structure/cell periphery |
| cassava4.1_021211m\|pacid:17976945 | -2.16 | -7.58 | -9.74 | Glucan endo-1,3-beta-glucosidase precursor | external encapsualting structure/cell periphery |
| cassava4.1_031387m\|pacid:17971806 | -8.23 | 5.39 | -2.84 | Xylan 1,4-beta-xylosidase | external encapsualting structure/cell periphery |
| cassava4.1_003768m\|pacid:17965079 | 1.63 | 0.48 | 2.11 | Hydrolase | external encapsualting structure/cell periphery |
| cassava4.1_003582m\|pacid:17969493 | -1.90 | 0.16 | -1.74 | Hydrolase, hydrolyzing O-glycosyl compounds | external encapsualting structure/cell periphery |
| cassava4.1_016458m\|pacid:17969209 | -1.32 | 0.18 | -1.14 | Hydrolase, hydrolyzing O-glycosyl compounds | cell periphery |
| cassava4.1_016746m\|pacid:17962714 | 1.73 | 0.57 | 2.30 | Hydrolase, hydrolyzing O-glycosyl compounds | external encapsualting structure |
| cassava4.1_002077m\|pacid:17962011 | 3.11 | -2.72 | 0.39 | Periplasmic beta-glucosidase precursor | external encapsualting structure/cell periphery |
| cassava4.1_014454m\|pacid:17988950 | -1.55 | 1.42 | -0.13 | Triosphosphate isomerase-like protein type II | external encapsualting structure/cell periphery |
| cassava4.1_004675m\|pacid:17977226 | 3.54 | -9.54 | -6.00 | Acid beta-fructofuranosidase precursor | external encapsualting structure/cell periphery |
| *Peptidase* | | | | | |
| cassava4.1_009061m\|pacid:17969679 | -1.15 | 1.02 | -0.13 | 26S protease regulatory subunit S10b | external encapsualting structure/cell periphery |
| cassava4.1_008061m\|pacid:17973239 | -6.70 | -5.43 | -12.13 | Aspartic proteinase nepenthesin-1 precursor | external encapsualting structure/cell periphery |
| cassava4.1_007900m\|pacid:17981909 | -3.79 | -8.80 | -12.59 | Aspartic proteinase nepenthesin-1 precursor | external encapsualting structure/cell periphery |
| cassava4.1_028838m\|pacid:17975752 | -3.44 | 1.47 | -1.97 | Aspartic proteinase nepenthesin-1 precursor | external encapsualting structure/cell periphery |
| cassava4.1_021811m\|pacid:17961238 | -8.32 | 8.32 | - | Serine carboxypeptidase | external encapsualting structure/cell periphery |
| cassava4.1_007203m\|pacid:17975343 | -2.83 | -2.93 | -5.76 | Serine carboxypeptidase | external encapsualting structure/cell periphery |
| cassava4.1_006631m\|pacid:17975345 | 3.15 | -2.60 | 0.55 | Serine carboxypeptidase | external encapsualting structure/cell periphery |
| cassava4.1_003895m\|pacid:17990740 | -1.77 | -0.63 | -2.40 | Xylem serine proteinase 1 precursor | external encapsualting structure/cell periphery |
| cassava4.1_002107m\|pacid:17992685 | -1.39 | -2.34 | -3.73 | Xylem serine proteinase 1 precursor | external encapsualting structure/cell periphery |
| cassava4.1_001801m\|pacid:17992095 | -1.13 | -0.56 | -1.69 | Peptidase | external encapsualting structure/cell periphery |
| *Stress response* | | | | | |
| cassava4.1_002964m\|pacid:17981435 | -2.42 | 1.18 | -1.24 | Heat shock protein | external encapsualting structure/cell periphery |
| cassava4.1_033681m\|pacid:17960765 | -1.09 | 2.08 | 0.99 | Heat shock protein | cell periphery |
| cassava4.1_002708m\|pacid:17989996 | -1.05 | 2.75 | 1.71 | Heat shock protein | external encapsualting structure/cell periphery |
| cassava4.1_021659m\|pacid:17991842 | 1.30 | -0.63 | 0.67 | Betaine-aldehyde dehydrogenase | external encapsualting structure/cell periphery |
| cassava4.1_020556m\|pacid:17960586 | -8.23 | - | -8.23 | Low-molecular-weight cysteine-rich 69 | external encapsualting structure/cell periphery |
| cassava4.1_004448m\|pacid:17991871 | -8.42 | - | -8.42 | Multicopper oxidase | external encapsualting structure/cell periphery |
| cassava4.1_020080m\|pacid:17990170 | -2.92 | 0.15 | -2.77 | Multicopper oxidase | cell periphery |
| cassava4.1_004981m\|pacid:17973582 | -1.97 | -1.50 | -3.47 | Multicopper oxidase | external encapsualting structure |
| cassava4.1_004139m\|pacid:17964554 | -1.76 | 0.77 | -0.99 | Multicopper oxidase | external encapsualting structure/cell periphery |
| cassava4.1_004947m\|pacid:17964024 | -1.11 | 0.37 | -0.74 | Multicopper oxidase | external encapsualting structure/cell periphery |
| cassava4.1_032882m\|pacid:17988105 | -8.12 | - | -8.12 | Peroxidase 12 precursor | external encapsualting structure/cell periphery |
| cassava4.1_012020m\|pacid:17989861 | -2.52 | 1.26 | -1.26 | Peroxidase 31 precursor | external encapsualting structure/cell periphery |
| cassava4.1_003278m\|pacid:17990275 | -9.85 | - | -9.85 | LRX2 | external encapsualting structure/cell periphery |
| *Cytoskeleton* | | | | | |
| cassava4.1_031429m\|pacid:17973711 | 8.66 | 0.64 | 9.30 | Fimbrin | external encapsualting structure/cell periphery |
| cassava4.1_006815m\|pacid:17975679 | -8.23 | 7.85 | -0.38 | PREDICTED: similar to TUBG1 (GAMMA-TUBULIN) | external encapsualting structure/cell periphery |
| cassava4.1_010649m\|pacid:17978498 | -1.96 | 1.36 | -0.60 | PREDICTED: similar to TUBG1 (GAMMA-TUBULIN) | external encapsualting structure |
| cassava4.1_007513m\|pacid:17961628 | 1.09 | -0.08 | 1.01 | tubulin alpha chain | external encapsualting structure/cell periphery |
| cassava4.1_007551m\|pacid:17965893 | 2.34 | 0.46 | 2.80 | Tubulin alpha-8 chain | external encapsualting structure/cell periphery |

Note: All data are shown in log_2_ratio, and the positive and negative values of log_2_ratio are either up- or downregulated genes in the three paired comparisons. No significant fold changes are indicated as “–”.
