# Supplementary material for: Transition from somatic embryo to friable embryogenic callus in cassava: dynamic changes in cellular structure, physiological status, and gene expression profiles
Source: Front Plant Sci. 2015 Oct 6;6:824. doi: 10.3389/fpls.2015.00824 (PMC4594424; doi:10.3389/fpls.2015.00824)
Supplement: Supplementary file 3 [file Table3.DOCX]

| **Supplementary Table 3.** DEGs involved in pathway of ‘Plant signal transduction’ | | | | | |
| --- | --- | --- | --- | --- | --- |
| Genes | FFEC  /SEs | OFEC  /FFEC | OFEC  /SEs | Blast nr | Description |
| cassava4.1_005565m\|pacid:17987922 | -2.03 | -0.12 | -2.16 | gi\|255538442 | Amino acid transporter |
| cassava4.1_007635m\|pacid:17972327 | -9.12 | 5.98 | -3.14 | gi\|255543198 | ATP binding protein |
| cassava4.1_032116m\|pacid:17990514 | -2.26 | -3.19 | -5.45 | gi\|255584451 | ATP binding protein |
| cassava4.1_003193m\|pacid:17978024 | -1.72 | -0.22 | -1.94 | gi\|255585468 | ATP binding protein |
| cassava4.1_003459m\|pacid:17979294 | -1.59 | -0.29 | -1.88 | gi\|255580373 | ATP binding protein |
| cassava4.1_028125m\|pacid:17975594 | -1.45 | 1.23 | -0.22 | gi\|255548077 | ATP binding protein |
| cassava4.1_003190m\|pacid:17984282 | -1.12 | -0.77 | -1.90 | gi\|255538220 | ATP binding protein |
| cassava4.1_008715m\|pacid:17981566 | 1.84 | 0.18 | 2.02 | gi\|255567596 | ATP binding protein |
| cassava4.1_027659m\|pacid:17979155 | 1.91 | 0.14 | 2.05 | gi\|255571408 | ATP binding protein |
| cassava4.1_027269m\|pacid:17992916 | 2.64 | -0.48 | 2.17 | gi\|255555031 | ATP binding protein |
| cassava4.1_001509m\|pacid:17974372 | 3.03 | -1.11 | 1.92 | gi\|255544644 | ATP binding protein |
| cassava4.1_006312m\|pacid:17974639 | 3.29 | -2.18 | 1.11 | gi\|255555031 | ATP binding protein |
| cassava4.1_031464m\|pacid:17985640 | 8.00 | -0.15 | 7.85 | gi\|255567058 | ATP binding protein |
| cassava4.1_007093m\|pacid:17993020 | -9.01 | - | -9.01 | gi\|126217796 | Auxin influx transport protein |
| cassava4.1_006474m\|pacid:17992396 | -1.27 | -2.74 | -4.01 | gi\|126217792 | Auxin influx transport protein |
| cassava4.1_001964m\|pacid:17967457 | -1.69 | 1.81 | 0.12 | gi\|255540071 | Auxin response factor |
| cassava4.1_019292m\|pacid:17965665 | -10.63 | - | -10.63 | gi\|255580941 | Auxin-induced protein 22D |
| cassava4.1_026033m\|pacid:17982968 | -10.12 | 6.39 | -3.73 | gi\|255552971 | Auxin-induced protein AUX22 |
| cassava4.1_025619m\|pacid:17959754 | -8.33 | - | -8.33 | gi\|255552971 | Auxin-induced protein AUX22 |
| cassava4.1_015577m\|pacid:17986246 | -3.89 | 0.30 | -3.59 | gi\|255559923 | Auxin-induced protein AUX28 |
| cassava4.1_017737m\|pacid:17988622 | 1.32 | -0.14 | 1.18 | gi\|255541222 | Auxin-induced protein X10A |
| cassava4.1_016728m\|pacid:17983007 | -2.40 | -1.05 | -3.45 | gi\|255552973 | Auxin-responsive protein IAA1 |
| cassava4.1_012530m\|pacid:17961831 | -9.12 | - | -9.12 | gi\|255574879 | Auxin-responsive protein IAA13 |
| cassava4.1_012387m\|pacid:17993487 | -4.85 | -1.24 | -6.09 | gi\|255574879 | Auxin-responsive protein IAA13 |
| cassava4.1_013129m\|pacid:17971668 | -2.05 | 1.49 | -0.56 | gi\|255571182 | Auxin-responsive protein IAA13 |
| cassava4.1_014849m\|pacid:17989380 | 1.10 | -0.94 | 0.16 | gi\|255580939 | Auxin-responsive protein IAA16 |
| cassava4.1_011048m\|pacid:17984115 | -1.89 | -1.02 | -2.92 | gi\|255549904 | Auxin-responsive protein IAA27 |
| cassava4.1_016873m\|pacid:17993367 | -2.35 | -1.46 | -3.82 | gi\|255551959 | Auxin-responsive protein IAA4 |
| cassava4.1_016842m\|pacid:17962255 | -1.07 | -1.14 | -2.21 | gi\|255551959 | Auxin-responsive protein IAA4 |
| assava4.1_029587m\|pacid:17984111 | -2.36 | -0.10 | -2.45 | gi\|255549816 | Auxin-responsive protein IAA6 |
| cassava4.1_010966m\|pacid:17973105 | -1.81 | -0.54 | -2.35 | gi\|255549816 | Auxin-responsive protein IAA6 |
| cassava4.1_008933m\|pacid:17979976 | -2.23 | 2.44 | 0.21 | gi\|255567750 | Big map kinase/bmk |
| cassava4.1_003643m\|pacid:17992851 | -3.64 | 0.72 | -2.92 | gi\|255568858 | BRASSINOSTEROID INSENSITIVE 1-associated receptor kinase 1 precursor |
| cassava4.1_003667m\|pacid:17987536 | -2.83 | -8.22 | -11.05 | gi\|255584308 | BRASSINOSTEROID INSENSITIVE 1-associated receptor kinase 1 precursor |
| cassava4.1_004481m\|pacid:17975156 | 1.08 | -0.44 | 0.64 | gi\|255557731 | BRASSINOSTEROID INSENSITIVE 1-associated receptor kinase 1 precursor |
| cassava4.1_003530m\|pacid:17978096 | 1.99 | -0.92 | 1.07 | gi\|255540437 | BRASSINOSTEROID INSENSITIVE 1-associated receptor kinase 1 precursor |
| cassava4.1_034354m\|pacid:17988058 | 8.87 | -1.16 | 7.71 | gi\|255581476 | Carbohydrate binding protein |
| cassava4.1_011423m\|pacid:17984399 | 3.89 | -1.34 | 2.55 | gi\|255569153 | Catalytic |
| cassava4.1_006077m\|pacid:17977068 | -8.01 | - | -8.01 | gi\|255568605 | Chitin-inducible gibberellin-responsive protein |
| cassava4.1_028155m\|pacid:17986059 | -3.38 | -5.43 | -8.81 | gi\|255568605 | Chitin-inducible gibberellin-responsive protein |
| cassava4.1_003937m\|pacid:17966195 | -2.39 | -1.26 | -3.65 | gi\|255561178 | Chitin-inducible gibberellin-responsive protein |
| cassava4.1_026197m\|pacid:17992689 | -3.85 | -5.43 | -9.28 | gi\|255538076 | Cyclin d |
| cassava4.1_010271m\|pacid:17968692 | -2.43 | -0.16 | -2.59 | gi\|255538192 | Cyclin d |
| cassava4.1_009900m\|pacid:17984979 | -2.02 | -2.51 | -4.54 | gi\|255555331 | Cyclin d |
| cassava4.1_009919m\|pacid:17984272 | -1.06 | -1.55 | -2.61 | gi\|255538192 | Cyclin d |
| cassava4.1_010382m\|pacid:17980567 | -2.26 | 1.27 | -0.99 | gi\|44889865 | Cyclin D3-2 |
| cassava4.1_003741m\|pacid:17987513 | -5.37 | -1.24 | -6.61 | gi\|255586838 | DELLA protein GAI |
| cassava4.1_005357m\|pacid:17981104 | -2.87 | -7.41 | -10.28 | gi\|255568267 | DELLA protein GAIP-B |
| cassava4.1_033968m\|pacid:17969699 | -2.01 | -1.84 | -3.85 | gi\|255573746 | DELLA protein GAIP-B |
| cassava4.1_011286m\|pacid:17989838 | -9.01 | - | -9.01 | gi\|255575991 | DNA binding protein |
| cassava4.1_009666m\|pacid:17992652 | -1.75 | -1.02 | -2.76 | gi\|255562645 | DNA binding protein |
| cassava4.1_007430m\|pacid:17989363 | 1.76 | -1.29 | 0.47 | gi\|255561969 | DNA binding protein |
| cassava4.1_010322m\|pacid:17991725 | 2.53 | -2.29 | 0.24 | gi\|255586149 | DNA binding protein |
| cassava4.1_007772m\|pacid:17981052 | 3.14 | -0.10 | 3.04 | gi\|255555441 | DNA binding protein |
| cassava4.1_000348m\|pacid:17980437 | -1.52 | -0.56 | -2.08 | gi\|255556978 | Ethylene insensitive protein |
| cassava4.1_002150m\|pacid:17993500 | -1.33 | -0.51 | -1.84 | gi\|255576860 | Ethylene receptor |
| cassava4.1_003444m\|pacid:17981455 | 1.10 | -0.85 | 0.25 | gi\|255564264 | Ethylene receptor |
| cassava4.1_004424m\|pacid:17971261 | -1.73 | 0.70 | -1.02 | gi\|255544776 | ETHYLENE-INSENSITIVE3 protein |
| cassava4.1_013138m\|pacid:17978862 | 1.83 | -4.45 | -2.61 | gi\|255583191 | Ethylene-responsive transcription factor 1A |
| cassava4.1_015673m\|pacid:17968366 | -3.32 | -5.43 | -8.74 | gi\|255583194 | Ethylene-responsive transcription factor 1B |
| cassava4.1_010495m\|pacid:17967915 | 2.13 | -0.49 | 1.64 | gi\|255553969 | Gibberellin receptor GID1 |
| cassava4.1_029685m\|pacid:17964403 | 3.87 | 0.17 | 4.04 | gi\|255538372 | Gibberellin receptor GID1 |
| cassava4.1_011788m\|pacid:17967142 | -4.32 | 0.19 | -4.13 | gi\|255539278 | Glucan endo-1,3-beta-glucosidase precursor |
| cassava4.1_007157m\|pacid:17978955 | -3.63 | 0.20 | -3.43 | gi\|255538616 | Glucan endo-1,3-beta-glucosidase precursor |
| cassava4.1_005968m\|pacid:17964195 | -2.77 | 1.44 | -1.32 | gi\|255546283 | Glucan endo-1,3-beta-glucosidase precursor |
| cassava4.1_007302m\|pacid:17982190 | 1.05 | 0.98 | 2.04 | gi\|255536825 | Glucan endo-1,3-beta-glucosidase precursor |
| cassava4.1_007509m\|pacid:17982795 | 2.16 | 0.01 | 2.17 | gi\|255536825 | Glucan endo-1,3-beta-glucosidase precursor |
| cassava4.1_007570m\|pacid:17989141 | 3.68 | -1.25 | 2.43 | gi\|255580057 | Glucan endo-1,3-beta-glucosidase precursor |
| cassava4.1_021465m\|pacid:17968858 | 8.87 | 0.28 | 9.15 | gi\|255554861 | Glucan endo-1,3-beta-glucosidase precursor |
| cassava4.1_009548m\|pacid:17987782 | 2.28 | 0.17 | 2.45 | gi\|224117378 | GRAS family transcription factor |
| cassava4.1_004650m\|pacid:17961624 | 2.48 | -1.32 | 1.16 | gi\|224126261 | GRAS family transcription factor |
| cassava4.1_003246m\|pacid:17984853 | 1.01 | -2.27 | -1.25 | gi\|255567047 | GRR1 |
| cassava4.1_000859m\|pacid:17961472 | -2.10 | -1.02 | -3.12 | gi\|255573224 | Histidine kinase 1, 2, 3 plant |
| cassava4.1_000780m\|pacid:17977897 | 1.15 | -2.06 | -0.91 | gi\|255547688 | Histidine kinase 1, 2, 3 plant |
| cassava4.1_018285m\|pacid:17986208 | -1.76 | 0.79 | -0.98 | gi\|255567590 | Histidine-containing phosphotransfer protein |
| cassava4.1_016458m\|pacid:17969209 | -1.32 | 0.18 | -1.14 | gi\|255547211\| | Hydrolase, hydrolyzing O-glycosyl compounds |
| cassava4.1_016746m\|pacid:17962714 | 1.73 | 0.57 | 2.30 | gi\|255547211\| | Hydrolase, hydrolyzing O-glycosyl compounds |
| cassava4.1_003660m\|pacid:17988615 | 1.24 | -0.69 | 0.55 | gi\|147769566 | Hypothetical protein VITISV_020732 |
| cassava4.1_003815m\|pacid:17971742 | -5.74 | -1.41 | -7.16 | gi\|255543248 | Indole-3-acetic acid-amido synthetase GH3.17 |
| cassava4.1_004140m\|pacid:17994001 | -8.88 | - | -8.88 | gi\|255567939 | Indole-3-acetic acid-amido synthetase GH3.3 |
| cassava4.1_004446m\|pacid:17978572 | -1.71 | -0.87 | -2.58 | gi\|255586541 | Indole-3-acetic acid-amido synthetase GH3.5 |
| cassava4.1_003838m\|pacid:17973943 | -10.79 | 5.39 | -5.40 | gi\|255586158 | Indole-3-acetic acid-amido synthetase GH3.6 |
| cassava4.1_003826m\|pacid:17980787 | -4.15 | -8.00 | -12.15 | gi\|255586158 | Indole-3-acetic acid-amido synthetase GH3.6 |
| cassava4.1_018659m\|pacid:17974359 | -3.09 | -8.00 | -11.09 | gi\|255544460 | Indole-3-acetic acid-induced protein ARG7 |
| cassava4.1_027025m\|pacid:17960998 | -2.38 | -0.31 | -2.69 | gi\|255543905 | Indole-3-acetic acid-induced protein ARG7 |
| cassava4.1_018580m\|pacid:17968304 | -1.33 | -3.78 | -5.11 | gi\|255544640 | Indole-3-acetic acid-induced protein ARG7 |
| sava4.1_003466m\|pacid:17993307 | -8.01 | 8.30 | 0.30 | gi\|255542910 | Kelch repeat protein |
| cassava4.1_027318m\|pacid:17963129 | -8.81 | - | -8.81 | gi\|255567375 | Kinase |
| cassava4.1_025847m\|pacid:17968658 | -2.01 | -2.52 | -4.53 | gi\|255570340 | Kinase |
| cassava4.1_030745m\|pacid:17974887 | 2.74 | -1.61 | 1.14 | gi\|255562540 | Kinase |
| cassava4.1_021326m\|pacid:17978116 | 8.11 | -1.14 | 6.98 | gi\|255577779 | Kinase |
| cassava4.1_000982m\|pacid:17961705 | 1.67 | -0.97 | 0.70 | gi\|255542402 | Map3k delta-1 protein kinase |
| cassava4.1_001236m\|pacid:17990580 | -1.56 | 0.03 | -1.54 | gi\|255568426 | Nodulation receptor kinase precursor |
| cassava4.1_033587m\|pacid:17973064 | -9.63 | 5.39 | -4.24 | gi\|255560627 | Nodulation signaling pathway 1 protein |
| cassava4.1_034192m\|pacid:17964880 | 8.00 | -2.61 | 5.39 | gi\|255569712 | Phytosulfokine receptor precursor |
| cassava4.1_013723m\|pacid:17960301 | 1.31 | -3.67 | -2.36 | gi\|297381026 | Plastid jasmonates ZIM-domain protein |
| cassava4.1_007926m\|pacid:17972067 | -4.64 | 2.14 | -2.50 | gi\|255562560 | Protein kinase |
| cassava4.1_013069m\|pacid:17990083 | -2.78 | -6.73 | -9.51 | gi\|255539096 | Protein kinase |
| cassava4.1_003640m\|pacid:17962984 | 1.06 | 0.22 | 1.29 | gi\|255553227 | Protein kinase |
| cassava4.1_010525m\|pacid:17978717 | -1.14 | -0.83 | -1.97 | gi\|310582 | Protein kinase 3 |
| cassava4.1_003001m\|pacid:17979101 | -3.64 | 0.55 | -3.09 | gi\|255575479 | Protein kinase APK1A, chloroplast precursor |
| cassava4.1_009853m\|pacid:17976664 | -1.08 | -2.34 | -3.42 | gi\|255552602 | Protein kinase atmrk1 |
| cassava4.1_009047m\|pacid:17986257 | 1.56 | -0.46 | 1.10 | gi\|255567520 | Protein kinase atmrk1 |
| cassava4.1_031181m\|pacid:17970782 | -8.01 | - | -8.01 | gi\|255537777 | Protein phosphatase 2c |
| cassava4.1_004984m\|pacid:17976065 | -3.99 | -0.02 | -4.00 | gi\|255560709 | Protein phosphatase 2c |
| cassava4.1_005959m\|pacid:17982124 | -2.88 | -6.00 | -8.88 | gi\|255546071 | Protein phosphatase 2c |
| cassava4.1_006830m\|pacid:17992177 | -2.60 | -0.72 | -3.32 | gi\|255553839 | Protein phosphatase 2c |
| cassava4.1_008162m\|pacid:17960657 | -2.39 | -0.39 | -2.77 | gi\|255566460 | Protein phosphatase 2c |
| cassava4.1_015832m\|pacid:17979856 | -1.16 | 0.07 | -1.09 | gi\|255553671 | Protein phosphatase 2c |
| cassava4.1_009531m\|pacid:17965363 | 1.27 | -0.03 | 1.23 | gi\|255573107 | Protein phosphatase 2c |
| cassava4.1_009913m\|pacid:17972789 | 1.36 | -1.02 | 0.34 | gi\|255573107 | Protein phosphatase 2c |
| cassava4.1_009537m\|pacid:17978036 | 3.16 | -1.77 | 1.39 | gi\|255560357 | Protein phosphatase 2c |
| cassava4.1_010061m\|pacid:17979685 | 3.95 | 0.26 | 4.21 | gi\|255551859 | Protein phosphatase 2c |
| cassava4.1_028714m\|pacid:17963849 | 8.00 | 0.57 | 8.57 | gi\|255583129 | Protein phosphatase 2c |
| cassava4.1_033110m\|pacid:17971409 | 8.11 | - | -8.11 | gi\|255569078 | Protein phosphatase 2c |
| cassava4.1_013488m\|pacid:17973848 | 8.50 | -0.40 | 8.10 | gi\|255542816 | Protein phosphatase 2c |
| cassava4.1_015839m\|pacid:17966672 | -1.89 | 0.09 | -1.80 | gi\|255576651 | Protein phosphatase-2c |
| cassava4.1_012871m\|pacid:17985903 | -1.59 | 1.57 | -0.02 | gi\|255548654 | Protein phosphatase-2c |
| cassava4.1_009497m\|pacid:17977919 | 1.94 | -0.79 | 1.15 | gi\|255547560 | Protein phosphatase-2c |
| cassava4.1_030583m\|pacid:17963376 | -3.95 | 1.97 | -1.98 | gi\|298103724 | Putative B-type response regulator 21 |
| cassava4.1_006440m\|pacid:17991967 | -1.19 | -0.42 | -1.61 | gi\|255558866 | Receptor protein kinase |
| cassava4.1_005916m\|pacid:17966980 | -1.12 | -0.19 | -1.30 | gi\|255539170 | Receptor protein kinase |
| cassava4.1_029655m\|pacid:17990525 | -9.01 | 5.39 | -3.61 | gi\|255555553 | Receptor-kinase |
| cassava4.1_004301m\|pacid:17984837 | -1.24 | 0.26 | -0.98 | gi\|255559053 | Regulatory protein NPR1 |
| cassava4.1_022028m\|pacid:17969868 | -8.23 | - | -8.23 | gi\|224103265 | SAUR family protein |
| cassava4.1_000881m\|pacid:17985893 | -3.53 | 1.19 | -2.34 | gi\|255548630 | Sensor histidine kinase |
| cassava4.1_000469m\|pacid:17971238 | -2.36 | -0.58 | -2.94 | gi\|255584913 | Serine/threonine-protein kinase BRI1 |
| cassava4.1_001075m\|pacid:17992334 | -1.32 | -3.78 | -5.09 | gi\|255583590 | Serine/threonine-protein kinase PBS1 |
| cassava4.1_001094m\|pacid:17970867 | 3.85 | -0.79 | 3.06 | gi\|255556695 | Serine/threonine-protein kinase PBS1 |
| cassava4.1_021900m\|pacid:17979899 | -9.78 | 5.39 | -4.38 | gi\|255583264 | Serine-threonine protein kinase |
| cassava4.1_010547m\|pacid:17983069 | -8.59 | - | -8.59 | gi\|255555345 | Serine-threonine protein kinase |
| cassava4.1_003347m\|pacid:17970559 | -8.23 | 6.71 | -1.51 | gi\|255550772 | Serine-threonine protein kinase |
| cassava4.1_015963m\|pacid:17983381 | -8.23 | - | -8.23 | gi\|255543144 | Serine-threonine protein kinase |
| cassava4.1_013816m\|pacid:17984574 | -2.68 | -0.88 | -3.56 | gi\|255579539 | Serine-threonine protein kinase |
| cassava4.1_011740m\|pacid:17979928 | -2.25 | -1.90 | -4.14 | gi\|255583264 | Serine-threonine protein kinase |
| cassava4.1_015019m\|pacid:17982970 | 1.05 | -2.08 | -1.02 | gi\|255549992 | Serine-threonine protein kinase |
| cassava4.1_008532m\|pacid:17969538 | 1.43 | 0.10 | 1.52 | gi\|255549992 | Serine-threonine protein kinase |
| cassava4.1_021846m\|pacid:17985988 | 1.79 | -3.40 | -1.61 | gi\|255549990 | Serine-threonine protein kinase |
| cassava4.1_017805m\|pacid:17991563 | 8.22 | 0.42 | 8.64 | gi\|255569851 | Serine-threonine protein kinase |
| cassava4.1_004080m\|pacid:17964208 | 8.41 | -1.44 | 6.98 | gi\|255577869 | Serine-threonine protein kinase |
| cassava4.1_016176m\|pacid:17982318 | 9.66 | -0.74 | 8.92 | gi\|255543643 | Serine-threonine protein kinase |
| cassava4.1_000605m\|pacid:17985416 | 1.74 | -0.26 | 1.48 | gi\|255570521 | Threonine protein kinase |
| cassava4.1_011303m\|pacid:17974550 | 1.37 | -0.60 | 0.77 | gi\|255552916 | Threonine-protein kinase SAPK3 |
| cassava4.1_001264m\|pacid:17973305 | 8.58 | -1.60 | 6.98 | gi\|255549760 | Threonine-protein kinase-transforming protein raf |
| cassava4.1_001986m\|pacid:17969299 | -8.51 | - | -8.51 | gi\|255557909 | Transcription factor |
| cassava4.1_027692m\|pacid:17962415 | -3.55 | -0.48 | -4.03 | gi\|255582934 | Transcription factor |
| cassava4.1_001737m\|pacid:17966005 | -2.10 | -0.70 | -2.79 | gi\|255557909 | Transcription factor |
| cassava4.1_002216m\|pacid:17983384 | -1.96 | -0.24 | -2.20 | gi\|255543190 | Transcription factor |
| cassava4.1_010425m\|pacid:17965177 | -1.75 | -2.93 | -4.67 | gi\|255562888 | Transcription factor |
| cassava4.1_009872m\|pacid:17988299 | -1.68 | 1.54 | -0.14 | gi\|255575999 | Transcription factor |
| cassava4.1_012328m\|pacid:17967613 | 1.29 | -0.75 | 0.54 | gi\|255554128 | Transcription factor |
| cassava4.1_003253m\|pacid:17967790 | 1.86 | -2.48 | -0.62 | gi\|255586180 | Transcription factor |
| cassava4.1_006556m\|pacid:17976973 | 2.05 | -1.02 | 1.03 | gi\|255544460 | Transcription factor |
| cassava4.1_005340m\|pacid:17962785 | 8.50 | 0.29 | 8.79 | gi\|255547079 | Transcription factor BIM1 |
| cassava4.1_025203m\|pacid:17993730 | 3.63 | -3.08 | 0.55 | gi\|255583258 | Transcription factor HBP-1b(c1) |
| cassava4.1_003408m\|pacid:17992550 | -8.81 | 6.98 | -1.83 | gi\|255545892 | TRANSPORT INHIBITOR RESPONSE 1 protein |
| cassava4.1_023767m\|pacid:17973338 | -1.96 | 1.01 | -0.95 | gi\|255544001 | Two-component response regulator ARR8 |
| cassava4.1_002953m\|pacid:17972685 | -1.50 | -2.44 | -3.93 | gi\|255565035 | Two-component system sensor histidine kinase/response regulator |
| cassava4.1_002564m\|pacid:17961082 | 1.81 | 0.42 | 2.23 | gi\|255586762 | Ubiquitin-protein ligase |
| cassava4.1_008404m\|pacid:17962703 | 2.62 | 0.27 | 2.89 | gi\|255546959 | Ubiquitin-protein ligase |
| cassava4.1_013286m\|pacid:17993141 | -3.09 | 2.26 | -0.83 | gi\|255570533 | Xyloglucan endotransglucosylase/hydrolase protein 22 precursor |

Note: All data are shown in log_2_ratio, and the positive and negative values of log_2_ratio are either up- or downregulated genes in the three paired comparisons. No significant fold changes are indicated as “–”.
