# Supplementary material for: Transition from somatic embryo to friable embryogenic callus in cassava: dynamic changes in cellular structure, physiological status, and gene expression profiles
Source: Front Plant Sci. 2015 Oct 6;6:824. doi: 10.3389/fpls.2015.00824 (PMC4594424; doi:10.3389/fpls.2015.00824)
Supplement: Supplementary file 4 [file Table4.DOCX]

| **Supplementary Table 4.** DEGs involved in the process of ‘Base excision repair’, ‘DNA replication’, ‘Nucleotide excision repair’ and ‘Match repair’ | | | | | |
| --- | --- | --- | --- | --- | --- |
| Genes | Description | FFEC/SE | OFEC/FFEC | OFEC/SE | Involved Pathway |
| cassava4.1_010657m | Replication factor C, RFC | - | 8.40 | 8.40 | 2,3,4 |
| cassava4.1_013998m | Proliferating cell nuclear antigen, PCNA | 0.01 | 2.60 | 2.59 | 1,2,3,4 |
| cassava4.1_013423m | Replication factor A, RFA/RPA | 6.00 | 8.85 | 2.85 | 2,3,4 |

Note: All data are shown in log_2_ratio, and the positive and negative values of log_2_ratio are either up- or downregulated genes in the three paired comparisons. No significant fold changes are indicated as “–”. The number in the column Involved Pathway stands for different categories of GO analysis: 1, Base excision repair; 2, DNA replication; 3, Nucleotide excision repair; 4, Mismatch repair.
