# Supplementary material for: Transition from somatic embryo to friable embryogenic callus in cassava: dynamic changes in cellular structure, physiological status, and gene expression profiles
Source: Front Plant Sci. 2015 Oct 6;6:824. doi: 10.3389/fpls.2015.00824 (PMC4594424; doi:10.3389/fpls.2015.00824)
Supplement: Supplementary file 5 [file Table5.DOCX]

| **Supplementary Table 5.** DEGs involved in the process of ‘cell cycle related terms’ | | | | | | |
| --- | --- | --- | --- | --- | --- | --- |
| Genes | FFEC  /SEs | OFEC  /FFEC | OFEC  /SEs | Blast nr | Description | Pathway |
| cassava4.1_009135m\|pacid:17960417 | -2.25 | 2.48 | 0.23 | gi\|255582138 | ATP binding protein | Cell cycle/cell cycle process |
| cassava4.1_020446m\|pacid:17982936 | -2.23 | 2.44 | 0.21 | gi\|255567750 | Big map kinase/bmk | M phase/cell cycle/cell cycle process |
| cassava4.1_000382m\|pacid:17981193 | -0.14 | 1.12 | 0.97 | gi\|255550664 | Carboxy-terminal kinesin | Cell cycle/cell cycle process |
| cassava4.1_008059m\|pacid:17962699 | -0.54 | 2.28 | 1.73 | gi\|255550664 | Carboxy-terminal kinesin | Cell cycle/cell cycle process |
| cassava4.1_005830m\|pacid:17986346 | 2.32 | 1.63 | 3.95 | gi\|255569307 | CDD6 | Cell cycle |
| cassava4.1_005106m\|pacid:17981511 | -0.22 | 2.38 | 2.16 | gi\|255557891 | CDK | M phase/cell cycle/cell cycle process |
| cassava4.1_007861m\|pacid:17965335 | 1.58 | 2.70 | 4.27 | gi\|255539342 | CDK | M phase/cell cycle/cell cycle process |
| cassava4.1_005535m\|pacid:17991934 | -0.50 | 2.57 | 2.07 | gi\|255576542 | Condensin, putative | M phase/cell cycle/cell cycle process |
| cassava4.1_004285m\|pacid:17963557 | 1.04 | -1.31 | -0.27 | gi\|255538588 | COP9 complex subunit | Cell cycle/cell cycle process |
| cassava4.1_020277m\|pacid:17963903 | -0.63 | 2.19 | 1.56 | gi\|255558608 | Cyclin A | Cell cycle |
| cassava4.1_000940m\|pacid:17963892 | - | 8.30 | 8.30 | gi\|255552983 | Cyclin A | Cell cycle |
| cassava4.1_009943m\|pacid:17986522 | -6.74 | 8.40 | 1.66 | gi\|255538138 | Cyclin A | Cell cycle |
| cassava4.1_007567m\|pacid:17980260 | -1.47 | 1.64 | 0.16 | gi\|255539220 | Cyclin B | Cell cycle |
| cassava4.1_034180m\|pacid:17967235 | 2.18 | 1.93 | 4.11 | gi\|255552467 | Cyclin B | M phase/cell cycle/cell cycle process |
| cassava4.1_033927m\|pacid:17973653 | -1.32 | 1.08 | -0.24 | gi\|42362268 | Cyclin-dependent kinases regulatory subunit | Cell cycle |
| cassava4.1_012120m\|pacid:17969326 | -0.13 | 1.87 | 1.74 | gi\|90856236 | Cyclin-dependent kinases regulatory subunit | Cell cycle |
| cassava4.1_008933m\|pacid:17979976 | 0.36 | 1.86 | 2.21 | gi\|255559927 | F-box/leucine rich repeat protein | M phase/cell cycle/cell cycle process |
| cassava4.1_006225m\|pacid:17988789 | - | 8.30 | 8.30 | gi\|255555065 | Kinesin | M phase/cell cycle/cell cycle process |
| cassava4.1_000308m\|pacid:17963456 | -7.00 | 8.40 | 1.40 | gi\|255563647 | Kinesin | M phase/cell cycle/cell cycle process |
| cassava4.1_013998m\|pacid:17991890 | 0.75 | 1.57 | 2.32 | gi\|255576776 | Kinetochore protein nuf2 | M phase/cell cycle/cell cycle process |
| cassava4.1_012271m\|pacid:17962626 | -1.27 | 1.27 | - | gi\|255559446 | Leucine rich repeat-containing protein | M phase/cell cycle/cell cycle process |
| cassava4.1_010009m\|pacid:17966041 | 0.12 | 1.35 | 1.47 | gi\|255559446 | Leucine rich repeat-containing protein | M phase/cell cycle/cell cycle process |
| cassava4.1_011646m\|pacid:17976911 | -2.00 | 3.92 | 1.91 | gi\|255587410 | Microtubule associated protein xmap215 | Cell cycle/cell cycle process |
| cassava4.1_021504m\|pacid:17963095 | 6.00 | 3.25 | 9.25 | gi\|255567371 | Pentatricopeptide repeat-containing protein | Cell cycle/cell cycle process |
| cassava4.1_010144m\|pacid:17992683 | - | 2.60 | 2.60 | gi\|255558840 | Proliferating cell nuclear antigen | Cell cycle |
| cassava4.1_024769m\|pacid:17982028 | -0.52 | 3.02 | 2.45 | gi\|255536887 | Ribonucleoside-diphosphate reductase small chain | Cell cycle |
| cassava4.1_021394m\|pacid:17961601 | -7.59 | 8.40 | 0.80 | gi\|255570163 | SMG-7 | M phase/cell cycle/cell cycle process |
| cassava4.1_001056m\|pacid:17979984 | -0.09 | 1.91 | 1.81 | gi\|255565533 | Ukaryotic translation initiation factor 2c | M phase/cell cycle/cell cycle process |

Note: All data are shown in log_2_ratio, and the positive and negative values of log_2_ratio are either up- or downregulated genes in FFEC vs SEs. No significant fold changes are indicated by “-”.
