# Supplementary material for: Transition from somatic embryo to friable embryogenic callus in cassava: dynamic changes in cellular structure, physiological status, and gene expression profiles
Source: Front Plant Sci. 2015 Oct 6;6:824. doi: 10.3389/fpls.2015.00824 (PMC4594424; doi:10.3389/fpls.2015.00824)
Supplement: Supplementary file 6 [file Table6.DOC]

| **Supplementary Table 6** Transcription factors involved in FEC induction and subculture process | | | | | |
| --- | --- | --- | --- | --- | --- |
| Genes | FFEC/SEs | OFEC/FFEC | OFEC/SEs | Blast nr | Description |
| cassava4.1_021810m|pacid:17968768 | **5.39** | **8.32** | 0 | gi|255577112 | transcription factor |
| cassava4.1_005473m|pacid:17967367 | **-6** | **7.71** | 1.71 | gi|255540073 | Transcription factor ICE1 |
| cassava4.1_030919m|pacid:17989059 | **7.56** | **7.56** | 0 | gi|255541048 | Ethylene-responsive transcription factor |
| cassava4.1_023052m|pacid:17976742 | **-7.23** | **7.56** | 0.33 | gi|255573943 | r2r3-myb transcription factor |
| cassava4.1_011038m|pacid:17986272 | **5.98** | **7.39** | 0 | gi|255559953 | WRKY transcription factor |
| cassava4.1_006374m|pacid:17968821 | **-5.43** | **7.2** | 1.77 | gi|255571218 | transcription factor |
| cassava4.1_004978m|pacid:17982584 | **-5.43** | **7.2** | 0 | gi|255551004 | transcription factor |
| cassava4.1_015131m|pacid:17988469 | **-6** | **7.2** | 1.2 | gi|255562856 | transcription factor |
| cassava4.1_030700m|pacid:17962334 | **-5.43** | **7** | 0 | gi|255578025 | transcription factor |
| cassava4.1_028757m|pacid:17962501 | **-6** | **6.98** | 0.98 | gi|255584594 | Transcription factor RF2a |
| cassava4.1_025554m|pacid:17978391 | **-6.43** | **6.71** | 0 | gi|255542074 | transcription factor |
| cassava4.1_032125m|pacid:17961949 | **-6.74** | **6.71** | -0.03 | gi|255556428 | transcription factor |
| cassava4.1_024131m|pacid:17988212 | **-7** | **6.71** | -0.29 | gi|255572876 | GATA transcription factor |
| cassava4.1_024970m|pacid:17966004 | **6.39** | **6.39** | 0 | gi|255561935 | transcription factor |
| cassava4.1_032115m|pacid:17988210 | **-5.43** | **6.39** | 0.97 | gi|255572874 | GATA transcription factor |
| cassava4.1_027980m|pacid:17993817 | **-6** | **6.39** | 0.39 | gi|255567234 | transcription factor |
| cassava4.1_028214m|pacid:17969027 | **5.98** | **5.98** | 0 | gi|269314027 | MADS-box transcription factor 3 |
| cassava4.1_005544m|pacid:17961073 | **5.98** | **5.98** | 0 | gi|255575383 | transcription factor |
| cassava4.1_006940m|pacid:17989270 | **-5.43** | **5.98** | 0 | gi|255555107 | transcription factor |
| cassava4.1_030636m|pacid:17979814 | **-6.43** | **5.98** | -0.45 | gi|255537575 | r2r3-myb transcription factor |
| cassava4.1_011597m|pacid:17971271 | **-7.88** | **5.98** | 0 | gi|255544894 | r2r3-myb transcription factor |
| cassava4.1_028162m|pacid:17966239 | **-9.12** | **5.98** | -3.14 | gi|255561158 | GATA transcription factor |
| cassava4.1_010323m|pacid:17970148 | **-11** | **5.98** | **-5.03** | gi|255555947 | transcription factor |
| cassava4.1_023141m|pacid:17968697 | **6.39** | **5.43** | 0 | gi|255538180 | r2r3-myb transcription factor |
| cassava4.1_021785m|pacid:17990430 | **-6** | **5.43** | **0** | gi|224072757 | AP2 domain-containing transcription factor |
| cassava4.1_008636m|pacid:17974519 | **6.98** | **5.39** | 0 | gi|255568946 | transcription factor |
| cassava4.1_021994m|pacid:17985317 | **5.39** | **5.39** | 0 | gi|255539943 | transcription factor |
| cassava4.1_021298m|pacid:17977627 | **5.39** | **5.39** | 0 | gi|255554813 | WRKY transcription factor |
| cassava4.1_024248m|pacid:17978529 | **5.39** | **5.39** | 0 | gi|255541712 | WRKY transcription factor |
| cassava4.1_027662m|pacid:17985979 | **5.39** | **5.39** | 0 | gi|255548742 | WRKY transcription factor |
| cassava4.1_022750m|pacid:17980258 | **-5.43** | **5.39** | 0 | gi|154243317 | MADS box transcription factor |
| cassava4.1_033513m|pacid:17980453 | **-5.43** | **5.39** | -0.03 | gi|255556914 | Transcription factor Dp-1 |
| cassava4.1_007936m|pacid:17976836 | **-5.43** | **5.39** | -0.03 | gi|224132326 | transcription factor E2F |
| cassava4.1_010816m|pacid:17983558 | **-6** | **5.39** | 0 | gi|255573408 | transcription factor |
| cassava4.1_026095m|pacid:17989556 | **-6** | **5.39** | 0 | gi|255585125 | transcription factor |
| cassava4.1_000904m|pacid:17970759 | **-6** | **5.39** | -0.61 | gi|255555651 | transcription factor |
| cassava4.1_005912m|pacid:17974977 | **-6** | **5.39** | -0.61 | gi|255542140 | WRKY transcription factor |
| cassava4.1_018091m|pacid:17984008 | **-6.43** | **5.39** | 0 | gi|255563302 | MADS-box transcription factor |
| cassava4.1_016663m|pacid:17993256 | **-7.59** | **5.39** | -2.2 | gi|255542922 | transcription factor |
| cassava4.1_008721m|pacid:17982085 | **-7.59** | **5.39** | -2.2 | gi|255577193 | Transcription factor RF2a |
| cassava4.1_010397m|pacid:17964276 | **-8.51** | **5.39** | -3.12 | gi|255546417 | transcription factor |
| cassava4.1_013975m|pacid:17981855 | **-10.28** | **5.39** | -4.88 | gi|224063317 | MADS9, AGL24 mads-box transcription factor |
| cassava4.1_015454m|pacid:17992079 | **-10.83** | **5.39** | 0 | gi|224095810 | MIKC mads-box transcription factor |
| cassava4.1_012154m|pacid:17976708 | -2.32 | 3.97 | 1.65 | gi|255559703 | WRKY transcription factor |
| cassava4.1_033219m|pacid:17969191 | **8.5** | 3.94 | **12.44** | gi|255547389 | transcription factor |
| cassava4.1_011442m|pacid:17959798 | -0.02 | 2.79 | 2.78 | gi|255574129 | transcription factor |
| cassava4.1_026340m|pacid:17986296 | 0.58 | 2.76 | 3.35 | gi|255559342 | transcription factor |
| cassava4.1_029090m|pacid:17971023 | 2.73 | 2.57 | **5.3** | gi|255537735 | transcription factor |
| cassava4.1_025848m|pacid:17982483 | -0.68 | 2.57 | 1.88 | i|255563476 | ccaat-binding transcription factor subunit A |
| cassava4.1_000461m|pacid:17986960 | -2.01 | 2.3 | 0.3 | gi|255577352 | transcription factor |
| cassava4.1_024908m|pacid:17978505 | **5.43** | 2.14 | **7.56** | gi|255583804 | Ethylene-responsive transcription factor |
| cassava4.1_017907m|pacid:17984927 | -1.12 | 1.85 | 0.74 | gi|255564393 | Nuclear transcription factor Y subunit A-4 |
| cassava4.1_012931m|pacid:17974541 | -1.42 | 1.85 | 0.43 | gi|255544375 | r2r3-myb transcription factor |
| cassava4.1_033345m|pacid:17993684 | 1.68 | 1.61 | 3.29 | gi|255582666 | Transcription factor HBP-1b |
| cassava4.1_021485m|pacid:17982019 | 0.57 | 1.57 | 2.14 | gi|255551209 | transcription factor |
| cassava4.1_009872m|pacid:17988299 | -1.68 | 1.54 | -0.14 | gi|255575999 | transcription factor |
| cassava4.1_012644m|pacid:17989640 | -1 | 1.39 | 0.39 | gi|255569964 | Transcription factor BIM1 |
| cassava4.1_012347m|pacid:17986391 | -0.94 | 1.35 | 0.41 | gi|51557078 | MYB transcription factor |
| cassava4.1_014053m|pacid:17974346 | -1.53 | 1.26 | -0.27 | gi|255544598 | ccaat-binding transcription factor |
| cassava4.1_006257m|pacid:17981793 | 0.91 | 1.25 | 2.16 | gi|255564494 | transcription factor |
| cassava4.1_016628m|pacid:17977027 | 1.58 | 1.24 | 2.82 | gi|225435733 | TBP-ASSOCIATED FACTOR 7,general RNA polymerase II transcription factor |
| cassava4.1_005020m|pacid:17966828 | -1.1 | 1.24 | 0.14 | gi|224072801 | AP2 domain-containing transcription factor |
| cassava4.1_023410m|pacid:17976097 | 4.08 | 1.11 | **5.19** | gi|255581093 | transcription factor |
| cassava4.1_001573m|pacid:17990861 | **5.43** | 0.97 | **6.39** | gi|255560846 | transcription factor |
| cassava4.1_025655m|pacid:17985972 | 0.83 | 0.94 | 1.77 | gi|255548748 | transcription factor |
| cassava4.1_010819m|pacid:17971301 | 0.09 | 0.94 | 1.02 | gi|255544876 | Nuclear transcription factor Y subunit A-1 |
| cassava4.1_025208m|pacid:17984415 | -1.92 | 0.89 | -1.02 | gi|255563160 | transcription factor |
| cassava4.1_011897m|pacid:17974351 | **7** | 0.85 | **7.85** | gi|255544632 | r2r3-myb transcription factor |
| cassava4.1_011610m|pacid:17987967 | 0.12 | 0.82 | 0.93 | gi|255574163 | transcription factor |
| cassava4.1_031883m|pacid:17990078 | 1.96 | 0.78 | 2.74 | gi|255539100 | transcription factor |
| cassava4.1_021201m|pacid:17969661 | 0.32 | 0.75 | 1.07 | gi|255538812 | transcription factor |
| cassava4.1_010314m|pacid:17986115 | -1 | 0.71 | -0.29 | gi|255559691 | Transcription factor RF2b |
| cassava4.1_010457m|pacid:17982687 | -1.28 | 0.71 | -0.57 | gi|255580133 | transcription factor |
| cassava4.1_017769m|pacid:17987022 | -1.9 | 0.67 | -1.23 | gi|255572650 | transcription factor |
| cassava4.1_005600m|pacid:17969562 | -0.24 | 0.66 | 0.42 | gi|255557639 | Transcription factor IWS1 |
| cassava4.1_008611m|pacid:17961603 | -0.08 | 0.65 | 0.57 | gi|255547590 | WRKY transcription factor |
| cassava4.1_009495m|pacid:17989477 | -0.88 | 0.64 | -0.24 | gi|255583910 | protease m50 membrane-bound transcription factor site 2 protease |
| cassava4.1_034312m|pacid:17966422 | -1.22 | 0.63 | -0.59 | gi|255585965 | transcription factor |
| cassava4.1_023473m|pacid:17973175 | -0.96 | 0.57 | -0.39 | gi|255560719 | transcription factor |
| cassava4.1_016924m|pacid:17985384 | -1.86 | 0.47 | -1.39 | gi|224084378 | AP2/ERF domain-containing transcription factor |
| cassava4.1_005076m|pacid:17978845 | 1.65 | 0.45 | 2.11 | gi|255580789 | GATA transcription factor |
| cassava4.1_007518m|pacid:17982683 | -0.86 | 0.44 | -0.41 | gi|255551323 | transcription factor |
| cassava4.1_002516m|pacid:17966521 | 0.72 | 0.43 | 1.15 | gi|255575245 | transcription factor |
| cassava4.1_013130m|pacid:17990891 | -0.19 | 0.43 | 0.24 | gi|255563368 | GATA transcription factor |
| cassava4.1_013364m|pacid:17976014 | -0.83 | 0.39 | -0.44 | gi|255560677 | Nuclear transcription factor Y subunit A-3 |
| cassava4.1_015936m|pacid:17961131 | -1.22 | 0.38 | -0.84 | gi|255543795 | phd finger transcription factor |
| cassava4.1_015422m|pacid:17965082 | -1.56 | 0.37 | -1.2 | gi|255540215 | ccaat-binding transcription factor |
| cassava4.1_014529m|pacid:17988064 | -1.2 | 0.33 | -0.87 | gi|255564496 | transcription factor |
| cassava4.1_009336m|pacid:17978235 | 0.25 | 0.32 | 0.58 | gi|255547822 | transcription factor |
| cassava4.1_024901m|pacid:17966213 | 0.41 | 0.3 | 0.71 | gi|255561409 | transcription factor |
| cassava4.1_034361m|pacid:17983803 | 0.41 | 0.3 | 0.71 | gi|255560137 | transcription factor |
| cassava4.1_000984m|pacid:17970968 | -1.01 | 0.3 | -0.7 | gi|255578890 | transcription factor |
| cassava4.1_005340m|pacid:17962785 | **8.5** | 0.29 | **8.79** | gi|255547079 | Transcription factor BIM1 |
| cassava4.1_008330m|pacid:17991578 | -1.21 | 0.29 | -0.92 | gi|116734642 | Myb transcription factor |
| cassava4.1_016630m|pacid:17990022 | -0.47 | 0.28 | -0.19 | gi|255580862 | transcription factor |
| cassava4.1_032889m|pacid:17977315 | -0.48 | 0.28 | -0.2 | gi|255622103 | ccaat-binding transcription factor subunit A |
| cassava4.1_024604m|pacid:17992113 | -0.64 | 0.27 | -0.38 | gi|255579552 | TFIIH basal transcription factor complex subunit |
| cassava4.1_020513m|pacid:17969020 | -2.08 | 0.25 | -1.83 | gi|255547409 | transcription factor |
| cassava4.1_011111m|pacid:17990229 | 1.84 | 0.24 | 2.08 | gi|255567897 | r2r3-myb transcription factor |
| cassava4.1_010599m|pacid:17989166 | -1.25 | 0.24 | -1.02 | gi|255571053 | transcription factor |
| cassava4.1_009548m|pacid:17987782 | 2.28 | 0.17 | 2.45 | gi|224117378 | GRAS family transcription factor |
| cassava4.1_015098m|pacid:17972312 | -0.43 | 0.16 | -0.27 | gi|268374460 | bHLH1 transcription factor |
| cassava4.1_009459m|pacid:17971336 | -2.35 | 0.16 | -2.19 | gi|255544780 | transcription factor |
| cassava4.1_002038m|pacid:17969806 | 1.01 | 0.13 | 1.14 | gi|255582509 | transcription factor |
| cassava4.1_024194m|pacid:17972929 | -0.59 | 0.13 | -0.46 | gi|255540585 | transcription factor |
| cassava4.1_018779m|pacid:17959913 | 0.78 | 0.11 | 0.89 | gi|255548646 | transcription factor |
| cassava4.1_013201m|pacid:17978824 | -1.8 | 0.11 | -1.69 | gi|255566759 | transcription factor |
| cassava4.1_009240m|pacid:17990955 | -0.09 | 0.08 | -0.01 | gi|255552430 | transcription factor |
| cassava4.1_005578m|pacid:17966522 | -0.49 | 0.03 | -0.46 | gi|255561140 | RNA polymerase II transcription factor |
| cassava4.1_006718m|pacid:17973005 | -0.79 | 0.03 | -0.76 | gi|255544089 | transcription factor |
| cassava4.1_032341m|pacid:17984610 | -1.01 | 0.02 | -0.99 | gi|255555833 | transcription factor |
| cassava4.1_013593m|pacid:17975173 | 0.3 | -0.01 | 0.29 | gi|224123754 | ERF domain-containing transcription factor |
| cassava4.1_021542m|pacid:17987786 | **7.22** | -0.02 | **7.2** | gi|255578114 | WRKY transcription factor |
| cassava4.1_027146m|pacid:17993261 | -1.33 | -0.02 | -1.35 | gi|255543212 | AP2 domain transcription factor RAP2.3 |
| cassava4.1_025714m|pacid:17964831 | -1.84 | -0.02 | -1.86 | gi|255563232 | transcription factor |
| cassava4.1_002193m|pacid:17981196 | -2.56 | -0.02 | -2.58 | gi|255555865 | transcription factor |
| cassava4.1_022296m|pacid:17981885 | **5.43** | -0.03 | **5.39** | gi|255545898 | transcription facto |
| cassava4.1_012965m|pacid:17989253 | -1.64 | -0.05 | -1.69 | gi|255569661 | transcription factor |
| cassava4.1_015656m|pacid:17967118 | -0.61 | -0.06 | -0.67 | gi|255554771 | phd finger transcription factor |
| cassava4.1_004461m|pacid:17975117 | 0.11 | -0.13 | -0.02 | gi|255576310 | WRKY transcription factor |
| cassava4.1_004348m|pacid:17989729 | 0.3 | -0.14 | 0.16 | gi|255560596 | transcription factor |
| cassava4.1_013880m|pacid:17961871 | -3.81 | -0.14 | -3.95 | gi|55419648 | EREBP transcription factor ERF-2 |
| cassava4.1_001412m|pacid:17983216 | 0.07 | -0.17 | -0.1 | gi|255585376 | nuclear transcription factor, X-box binding |
| cassava4.1_002817m|pacid:17966371 | -0.88 | -0.17 | -1.05 | gi|255569375 | transcription factor |
| cassava4.1_000250m|pacid:17981887 | 0.66 | -0.18 | 0.49 | gi|255551243 | transcription factor |
| cassava4.1_018617m|pacid:17979670 | -0.37 | -0.18 | -0.55 | gi|255556286 | GATA transcription factor |
| cassava4.1_016130m|pacid:17963251 | **7.58** | -0.19 | **7.39** | gi|269314025 | MADS-box transcription factor 2 |
| cassava4.1_001408m|pacid:17965912 | -0.53 | -0.19 | -0.73 | gi|255575574 | transcription factor |
| cassava4.1_032473m|pacid:17978796 | **7.41** | -0.21 | **7.2** | gi|255587766 | Ethylene-responsive transcription factor |
| cassava4.1_021176m|pacid:17963761 | 3.32 | -0.22 | 3.1 | gi|255548239 | WRKY transcription factor |
| cassava4.1_011620m|pacid:17994055 | 2.13 | -0.23 | 1.9 | gi|255566171 | transcription factor |
| cassava4.1_022038m|pacid:17989010 | -0.72 | -0.23 | -0.95 | gi|255579124 | transcription factor |
| cassava4.1_002216m|pacid:17983384 | -1.96 | -0.24 | -2.2 | gi|255543190 | transcription factor |
| cassava4.1_017349m|pacid:17969659 | -0.24 | -0.25 | -0.49 | gi|302584056 | MADS domain transcription factor |
| cassava4.1_022504m|pacid:17963945 | -0.44 | -0.25 | -0.69 | gi|255577114 | transcription factor |
| cassava4.1_012252m|pacid:17992340 | -3.61 | -0.29 | -3.89 | gi|255579355 | transcription factor |
| cassava4.1_032811m|pacid:17987703 | 3.07 | -0.3 | 2.78 | gi|224131790 | GRAS family transcription factor |
| cassava4.1_001997m|pacid:17975019 | -1.12 | -0.32 | -1.44 | gi|255583321 | transcription factor |
| cassava4.1_017977m|pacid:17974422 | -1.26 | -0.32 | -1.58 | gi|295002526 | putative NAC transcription factor |
| cassava4.1_013591m|pacid:17971032 | -0.44 | -0.36 | -0.8 | gi|255537643 | transcription factor |
| cassava4.1_012485m|pacid:17980541 | -1.24 | -0.37 | -1.61 | gi|255543685 | transcription factor |
| cassava4.1_017103m|pacid:17969972 | -0.17 | -0.43 | -0.6 | gi|255563026 | Ethylene-responsive transcription factor |
| cassava4.1_030046m|pacid:17992563 | -1.6 | -0.43 | -2.03 | gi|255545906 | transcription factor |
| cassava4.1_027692m|pacid:17962415 | -3.55 | -0.48 | -4.03 | gi|255582934 | transcription factor |
| cassava4.1_017747m|pacid:17968029 | 1.06 | -0.54 | 0.52 | gi|255544452 | transcription factor |
| cassava4.1_019361m|pacid:17972572 | -0.13 | -0.57 | -0.7 | gi|255565846 | ccaat-binding transcription factor subunit A |
| cassava4.1_012556m|pacid:17963270 | **6** | -0.61 | **5.39** | gi|255541536 | r2r3-myb transcription factor |
| cassava4.1_021857m|pacid:17985170 | **6** | -0.61 | **5.39** | gi|255541106 | r2r3-myb transcription factor |
| cassava4.1_028473m|pacid:17962586 | **6** | -0.61 | **5.39** | gi|255587680 | Transcription factor ICE1 |
| cassava4.1_014812m|pacid:17972181 | 0.57 | -0.61 | -0.03 | gi|255575594 | WRKY transcription factor |
| cassava4.1_002918m|pacid:17983950 | -0.21 | -0.63 | -0.83 | gi|255557569 | Transcription factor AtMYC2 |
| cassava4.1_014105m|pacid:17962102 | -1.05 | -0.66 | -1.7 | gi|255554246 | GATA transcription factor |
| cassava4.1_017418m|pacid:17964035 | -0.42 | -0.67 | -1.1 | gi|255568424 | ccaat-binding transcription factor subunit A |
| cassava4.1_013174m|pacid:17992976 | -0.42 | -0.68 | -1.1 | gi|292698371 | heat stress transcription factor |
| cassava4.1_024052m|pacid:17970192 | 1.41 | -0.7 | 0.71 | gi|255569211 | transcription factor |
| cassava4.1_034188m|pacid:17962543 | -0.01 | -0.7 | -0.7 | gi|255576233 | transcription factor |
| cassava4.1_011390m|pacid:17989777 | -0.43 | -0.72 | -1.15 | gi|255568528 | transcription factor |
| cassava4.1_002147m|pacid:17961196 | -1.03 | -0.72 | -1.75 | gi|255570484 | transcription factor hy5 |
| cassava4.1_019256m|pacid:17971786 | 0.71 | -0.74 | -0.02 | gi|255545584 | Transcription factor TGA7 |
| cassava4.1_002197m|pacid:17967827 | -0.58 | -0.74 | -1.32 | gi|255586188 | transcription factor |
| cassava4.1_012328m|pacid:17967613 | 1.29 | -0.75 | 0.54 | gi|255554128 | transcription factor |
| cassava4.1_033113m|pacid:17992405 | **7.73** | -0.76 | **6.98** | gi|255574038 | WRKY transcription factor |
| cassava4.1_030395m|pacid:17980172 | 2.12 | -0.77 | 1.35 | gi|255567719 | WRKY transcription factor |
| cassava4.1_010007m|pacid:17968704 | 1.36 | -0.79 | 0.57 | gi|255538212 | WRKY transcription factor |
| cassava4.1_009108m|pacid:17982999 | 0.51 | -0.81 | -0.3 | gi|255574570 | transcription factor |
| cassava4.1_001576m|pacid:17984754 | -0.04 | -0.82 | -0.86 | gi|255565913 | transcription factor |
| cassava4.1_012449m|pacid:17988320 | -0.33 | -0.82 | -1.14 | gi|255558326 | transcription factor |
| cassava4.1_018715m|pacid:17993536 | **7.22** | -0.83 | **6.39** | gi|255576812 | Ethylene-responsive transcription factor |
| cassava4.1_011135m|pacid:17960892 | 0.48 | -0.83 | -0.35 | gi|255548594 | WRKY transcription factor |
| cassava4.1_003039m|pacid:17972978 | 2.06 | -0.84 | 1.22 | gi|171452362 | transcription factor |
| cassava4.1_020578m|pacid:17975948 | -0.26 | -0.87 | -1.13 | gi|255576983 | WRKY transcription factor |
| cassava4.1_034078m|pacid:17970895 | 1.87 | -0.89 | 0.98 | gi|255555685 | r2r3-myb transcription factor |
| cassava4.1_008041m|pacid:17988085 | 1.69 | -0.91 | 0.77 | gi|255586449 | WRKY transcription factor |
| cassava4.1_001357m|pacid:17970373 | -0.52 | -0.93 | -1.46 | gi|302398563 | ARF domain class transcription factor |
| cassava4.1_011097m|pacid:17969449 | 1.92 | -0.98 | 0.94 | gi|259121417 | WRKY transcription factor 26 |
| cassava4.1_006556m|pacid:17976973 | 2.05 | -1.02 | 1.03 | gi|255544460 | transcription factor |
| cassava4.1_017377m|pacid:17965024 | 0.57 | -1.02 | -0.45 | gi|255540179 | WRKY transcription factor |
| cassava4.1_016658m|pacid:17980506 | -1.33 | -1.02 | -2.35 | gi|255580369 | ccaat-binding transcription factor subunit A |
| cassava4.1_004933m|pacid:17968031 | 1.3 | -1.03 | 0.27 | gi|255546686 | transcription factor |
| cassava4.1_022469m|pacid:17981733 | -1.25 | -1.03 | -2.28 | gi|255583340 | transcription factor |
| cassava4.1_001151m|pacid:17966611 | -1.22 | -1.05 | -2.27 | gi|255542696 | transcription factor |
| cassava4.1_011936m|pacid:17964045 | **8.11** | -1.14 | **6.98** | gi|255557405 | WRKY transcription factor |
| cassava4.1_032899m|pacid:17966543 | 0.78 | -1.16 | -0.38 | gi|255561122 | transcription factor |
| cassava4.1_005571m|pacid:17964618 | -1.55 | -1.16 | -2.71 | gi|255548111 | transcription factor |
| cassava4.1_002957m|pacid:17968543 | -0.19 | -1.19 | -1.38 | gi|224084478 | AP2 domain-containing transcription factor |
| cassava4.1_007480m|pacid:17966757 | -0.49 | -1.24 | -1.73 | gi|255544826 | transcription factor |
| cassava4.1_006539m|pacid:17985377 | -0.84 | -1.24 | -2.08 | gi|224096732 | AP2 domain-containing transcription factor |
| cassava4.1_029459m|pacid:17965309 | -0.16 | -1.25 | -1.41 | gi|255588039 | r2r3-myb transcription factor |
| cassava4.1_012307m|pacid:17990308 | -1.56 | -1.26 | -2.83 | gi|255581935 | transcription factor |
| cassava4.1_007151m|pacid:17972557 | 0.12 | -1.28 | -1.17 | gi|224109656 | AP2 domain-containing transcription factor |
| cassava4.1_014600m|pacid:17992468 | -1.42 | -1.28 | -2.7 | gi|255576577 | transcription factor |
| cassava4.1_004650m|pacid:17961624 | 2.48 | -1.32 | 1.16 | gi|224126261 | GRAS family transcription factor |
| cassava4.1_023136m|pacid:17963756 | **6.73** | -1.34 | **5.39** | gi|255548357 | r2r3-myb transcription factor |
| cassava4.1_005267m|pacid:17983584 | -0.27 | -1.34 | -1.61 | gi|255578314 | WRKY transcription factor |
| cassava4.1_016275m|pacid:17985281 | 0.99 | -1.46 | -0.46 | gi|224057956 | AP2/ERF domain-containing transcription factor |
| cassava4.1_002125m|pacid:17967766 | 0.12 | -1.47 | -1.35 | gi|255586186 | transcription factor |
| cassava4.1_011430m|pacid:17961128 | -1.55 | -1.54 | -3.09 | gi|255543845 | GATA transcription factor |
| cassava4.1_014035m|pacid:17978188 | -0.2 | -1.71 | -1.9 | gi|255562482 | WRKY transcription factor |
| cassava4.1_012593m|pacid:17963731 | -1.26 | -1.71 | -2.97 | gi|255548389 | WRKY transcription factor |
| cassava4.1_019891m|pacid:17965521 | 2.37 | -1.8 | 0.56 | gi|302398709 | C3HL domain class transcription factor |
| cassava4.1_014624m|pacid:17973971 | 2.94 | -1.82 | 1.12 | gi|255547411 | r2r3-myb transcription factor |
| cassava4.1_008256m|pacid:17960343 | -2.1 | -1.89 | -4 | gi|255571722 | transcription factor |
| cassava4.1_007074m|pacid:17973059 | 0.26 | -1.9 | -1.64 | gi|255543929 | Transcription factor HBP-1b(c1) |
| cassava4.1_027917m|pacid:17992063 | **9.32** | -1.93 | **7.39** | gi|255552876 | WRKY transcription factor |
| cassava4.1_009059m|pacid:17964295 | 3.9 | -1.93 | 1.97 | gi|255571505 | transcription factor |
| cassava4.1_026346m|pacid:17968792 | -1.93 | -1.93 | -3.86 | gi|255578141 | GATA transcription factor |
| cassava4.1_014435m|pacid:17976610 | **8.11** | -2.14 | **5.98** | gi|255579979 | r2r3-myb transcription factor |
| cassava4.1_003030m|pacid:17973354 | -0.64 | -2.19 | -2.84 | gi|255582403 | transcription factor |
| cassava4.1_023697m|pacid:17980763 | -1.76 | -2.24 | -4 | gi|255536755 | Ethylene-responsive transcription factor |
| cassava4.1_014721m|pacid:17970026 | 3.23 | -2.4 | 0.82 | gi|255536751 | Ethylene-responsive transcription factor |
| cassava4.1_032424m|pacid:17979862 | -0.67 | -2.44 | -3.11 | gi|255553661 | Ethylene-responsive transcription factor |
| cassava4.1_033707m|pacid:17970844 | -0.06 | -2.45 | -2.51 | gi|255586369 | AP2 domain transcription factor RAP2.3 |
| cassava4.1_003253m|pacid:17967790 | 1.86 | -2.48 | -0.62 | gi|255586180 | transcription factor |
| cassava4.1_032848m|pacid:17969616 | 1.13 | -2.48 | -1.35 | gi|255565737 | transcription factor |
| cassava4.1_011894m|pacid:17961853 | 0.79 | -2.48 | -1.69 | gi|255556432 | WRKY transcription factor |
| cassava4.1_010425m|pacid:17965177 | -1.75 | -2.93 | -4.67 | gi|255562888 | transcription factor |
| cassava4.1_010191m|pacid:17964717 | 2.36 | -2.97 | -0.61 | gi|255568994 | transcription factor |
| cassava4.1_025203m|pacid:17993730 | 3.63 | -3.08 | 0.55 | gi|255583258 | Transcription factor HBP-1b(c1) |
| cassava4.1_010367m|pacid:17988694 | 0.8 | -3.1 | -2.29 | gi|224083717 | ERF domain-containing transcription factor |
| cassava4.1_016343m|pacid:17965263 | **9.94** | -3.22 | **6.71** | gi|224069196 | AP2/ERF domain-containing transcription factor |
| cassava4.1_013315m|pacid:17964587 | 0.23 | -3.37 | -3.14 | gi|224112801 | ERF domain-containing transcription factor |
| cassava4.1_011029m|pacid:17963298 | 2.38 | -3.41 | -1.03 | gi|255557623 | transcription factor |
| cassava4.1_023502m|pacid:17977929 | 1.99 | -3.76 | -1.76 | gi|255547662 | WRKY transcription factor |
| cassava4.1_026701m|pacid:17988332 | 2.35 | -4.19 | -1.84 | gi|255558194 | WRKY transcription factor |
| cassava4.1_013138m|pacid:17978862 | 1.83 | -4.45 | -2.61 | gi|255583191 | Ethylene-responsive transcription factor 1A |
| cassava4.1_026359m|pacid:17989607 | **5.98** | **-5.43** | 0 | gi|255574310 | ccaat-binding transcription factor |
| cassava4.1_031623m|pacid:17970526 | **5.43** | **-5.43** | 0 | gi|224088649 | AP2/ERF domain-containing transcription factor |
| cassava4.1_028415m|pacid:17960906 | **5.43** | **-5.43** | 0 | gi|255574095 | transcription factor |
| cassava4.1_030626m|pacid:17965612 | **5.43** | **-5.43** | 0 | gi|255558033 | transcription factor |
| cassava4.1_013408m|pacid:17972968 | 0 | **-5.43** | **-5.43** | gi|255574826 | transcription factor |
| cassava4.1_011813m|pacid:17988628 | -0.57 | **-5.43** | **-6** | gi|255582532 | Transcription factor TGA1 |
| cassava4.1_022241m|pacid:17984123 | -1 | **-5.43** | **-6.43** | gi|255549860 | GATA transcription factor |
| cassava4.1_015180m|pacid:17985331 | **-1.57** | **-5.43** | **-7** | gi|224057954 | AP2/ERF domain-containing transcription factor |
| cassava4.1_022538m|pacid:17966184 | -2.8 | **-5.43** | **-8.23** | gi|255563028 | Ethylene-responsive transcription factor |
| cassava4.1_018662m|pacid:17987234 | -2.99 | **-5.43** | **-8.42** | gi|255583196 | Ethylene-responsive transcription factor |
| cassava4.1_015673m|pacid:17968366 | -3.32 | **-5.43** | **-8.74** | gi|255583194 | Ethylene-responsive transcription factor 1B |
| cassava4.1_020929m|pacid:17960864 | -3.38 | **-5.43** | **-8.81** | gi|255545072 | Ethylene-responsive transcription factor |
| cassava4.1_010811m|pacid:17968727 | **-5.43** | **-5.43** | 0 | gi|255586867 | GATA transcription factor |
| cassava4.1_010627m|pacid:17986737 | **-5.43** | **-5.43** | 0 | gi|255553570 | Nuclear transcription factor Y subunit A-1 |
| cassava4.1_013112m|pacid:17964800 | **-5.43** | **-5.43** | 0 | gi|255569812 | transcription factor |
| cassava4.1_026876m|pacid:17970254 | **-5.43** | **-5.43** | 0 | gi|255581025 | transcription factor |
| cassava4.1_012637m|pacid:17992307 | **-5.43** | **-5.43** | 0 | gi|193237557 | transcription factor CCAAT |
| cassava4.1_027443m|pacid:17968247 | **-6** | **-5.43** | 0 | gi|111218108 | DREB-like transcription factor 1 |
| cassava4.1_011576m|pacid:17990675 | **6.98** | **-6** | 0 | gi|255546029 | Nuclear transcription factor Y subunit A-3 |
| cassava4.1_017987m|pacid:17974393 | **6** | **-6** | 0 | gi|255568693 | phd finger transcription factor |
| cassava4.1_022027m|pacid:17976459 | -0.43 | **-6** | **-6.43** | gi|224074209 | AP2/ERF domain-containing transcription factor |
| cassava4.1_034303m|pacid:17967578 | -0.74 | **-6** | **-6.74** | gi|255580742 | Ethylene-responsive transcription factor |
| cassava4.1_033153m|pacid:17981902 | -0.74 | **-6** | **-6.74** | gi|224059476 | GRAS family transcription factor |
| cassava4.1_025768m|pacid:17967628 | **-5.43** | **-6** | 0 | gi|255554110 | transcription factor |
| cassava4.1_023237m|pacid:17985249 | **-6** | **-6** | 0 | gi|308154466 | palmate-like pentafoliata 1 transcription factor |
| cassava4.1_014820m|pacid:17979093 | **-6.43** | **-6** | 0 | gi|255575477 | transcription factor |
| cassava4.1_015856m|pacid:17993557 | -0.33 | **-6.41** | **-6.74** | gi|255581463 | Ethylene-responsive transcription factor 1B |
| cassava4.1_013969m|pacid:17973327 | **-6.43** | **-6.43** | 0 | gi|255543975 | transcription factor |
| cassava4.1_003817m|pacid:17966840 | **5.39** | **-7** | 0 | gi|255544886 | transcription factor |
| cassava4.1_028977m|pacid:17993693 | 0.26 | **-7** | **-6.74** | gi|255537591 | WRKY transcription factor |
| cassava4.1_034370m|pacid:17960098 | -1.67 | **-7** | **-8.67** | gi|255583354 | transcription factor |
| cassava4.1_010769m|pacid:17968618 | -0.37 | **-7.22** | **-7.59** | gi|33338106 | transcription factor DP1 |
| cassava4.1_026705m|pacid:17960934 | -2.37 | **-7.22** | **-9.59** | gi|224063112 | ERF domain-containing transcription factor |
| cassava4.1_022816m|pacid:17969970 | -0.71 | **-7.41** | **-8.12** | gi|224077026 | AP2/ERF domain-containing transcription factor |
| cassava4.1_029658m|pacid:17975269 | 1.58 | **-7.58** | **-6** | gi|224053406 | AP2/ERF domain-containing transcription factor |
| cassava4.1_008770m|pacid:17962842 | -0.16 | **-7.58** | **-7.74** | gi|255576507 | Transcription factor HBP-1b(c1) |
| cassava4.1_031672m|pacid:17977328 | -2.08 | **-7.58** | **-9.67** | gi|255538274 | transcription factor |
| cassava4.1_003646m|pacid:17975739 | -1.33 | **-7.73** | **-9.06** | gi|255541590 | transcription factor |
| cassava4.1_032017m|pacid:17977240 | **-8.01** | **-8.01** | 0 | gi|255577819 | WRKY transcription factor |
| cassava4.1_004464m|pacid:17987916 | **7.39** | **-8.12** | 0 | gi|255538406 | transcription factor |
| cassava4.1_025934m|pacid:17965298 | **-8.12** | **-8.12** | 0 | gi|255588035 | transcription factor |
| cassava4.1_001986m|pacid:17969299 | **-8.51** | **-8.51** | 0 | gi|255557909 | transcription factor |
| cassava4.1_010602m|pacid:17966133 | -2.22 | **-8.66** | **-10.88** | gi|259121415 | WRKY transcription factor |
| cassava4.1_018746m|pacid:17971467 | **-7.74** | **-10.37** | 0 | gi|255551711 | ccaat-binding transcription factor subunit A |
| cassava4.1_015575m|pacid:17980651 | **-11.63** | **-11.63** | 0 | gi|40806814 | MADS box transcription factor |
| cassava4.1_031758m|pacid:17980817 | **5.39** | **-12.81** | 0 | gi|255543647 | transcription factor |
| Note: All data are shown in log_2_ratio, and the positive and negative values of log_2_ratio are either up- or downregulated genes. The log2ratio values greater than five were colored red, and those with negative values colored blue. The order is based on the result of OFEC/FFEC. | | | | | |
