# Supplementary figures and images for: Transition from somatic embryo to friable embryogenic callus in cassava: dynamic changes in cellular structure, physiological status, and gene expression profiles
Source: Front Plant Sci. 2015 Oct 6;6:824. doi: 10.3389/fpls.2015.00824 (PMC4594424; doi:10.3389/fpls.2015.00824)

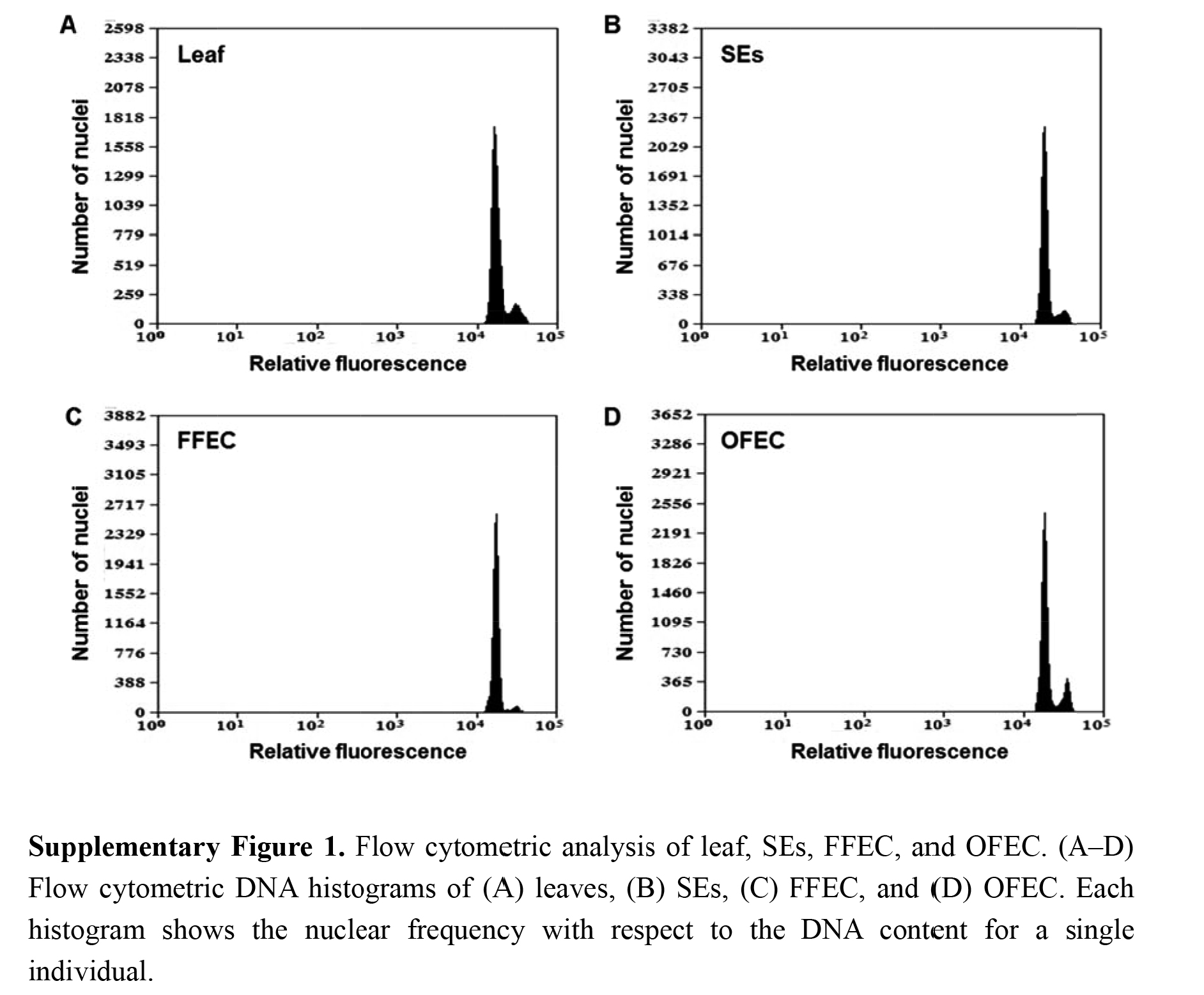

Supplement: Supplementary file 7 [file Image1.JPEG]
